# Quantifying Diagnostic Misclassification and Inpatient Mortality Determinants in Scrub Typhus: A Systematic Review and Interaction Meta-Analysis of Acute Undifferentiated Febrile Illness (AUFI) Cohorts in India

**DOI:** 10.7759/cureus.116141

**Published:** 2026-09-12

**Authors:** Jayashankar C. A., Venkataramana Kandi, Ganaraja V Harikrishna, Koshy T Sam, Manish G. R., Atul S Sucharitha, Divyadharshini M. S., Praful Bhupathiraju, Satya Vijaya Chandana Batchu, Bhangdiya G Sanjay, Sudishna M S.

**Affiliations:** 1 Internal Medicine, Vydehi Institute of Medical Sciences and Research Centre, Bengaluru, IND; 2 Clinical Microbiology, Prathima Institute of Medical Sciences, Nagunoor, IND; 3 Neurology, National Institute of Mental Health and Neuro Sciences, Bengaluru, IND; 4 General Medicine, Akash Institute of Medical Sciences and Research Centre, Bengaluru, IND; 5 General Medicine, Vydehi Institute of Medical Sciences and Research Centre, Bengaluru, IND; 6 Internal Medicine, Manipal Hospital, Bengaluru, IND

**Keywords:** acute undifferentiated febrile illness (aufi), diagnostic bias, india, meta-analysis, mortality risk, orientia tsutsugamushi, prevalence, scrub typhus

## Abstract

Scrub typhus (ST), caused by *Orientia tsutsugamushi*,* *is an underrecognized but major etiology of acute undifferentiated febrile illness (AUFI) across India. Prior national syntheses were limited by selection biases arising from heterogeneous screening denominators. To establish an unbiased epidemiological baseline, we conducted a Preferred Reporting Items for Systematic Reviews and Meta‑Analyses‑compliant systematic review and meta‑analysis (registered with the International Prospective Register of Systematic Reviews) of observational studies published between January 2015 and June 2026, enforcing strict AUFI syndromic gatekeeping. Sixteen institutional cohorts comprising 27,892 screened AUFI patients met inclusion criteria. DerSimonian-Laird random‑effects modeling was applied to estimate pooled prevalence and heterogeneity. The pooled prevalence of ST was 14.60% (95% confidence interval (CI): 11.27-18.71; I^2 ^= 97.40%, p < 0.001), with marked regional disparities: East/Central India 22.70% (95% CI: 16.80-29.80), South India 16.70% (95% CI: 10.20-25.90), North India 13.80% (95% CI: 8.40-21.50), and West India 2.50% (95% CI: 0.80-7.20; p < 0.001). The nationwide 95% prediction interval (3.30-45.20) captured extreme localized microfocal transmission dynamics. Multivariable meta‑regression explained 68.90% of between‑study variance (R^2 ^= 68.90%; residual I^2 ^= 34.20%), identifying a significant interaction between diagnostic assay stringency and regional transmission intensity (β = 0.485, p = 0.041). Cohorts employing high‑rigor composite criteria, including immunoglobulin M (IgM) enzyme‑linked immunosorbent assay (ELISA), polymerase chain reaction (PCR), indirect immunofluorescence assay, or eschar confirmation, reported a pooled prevalence of 17.40% (95% CI: 12.80-23.10), compared with 11.90% (95% CI: 7.60-18.00) in cohorts using single‑modality rapid immunochromatographic tests (ICTs) or legacy Weil-Felix assays (p = 0.024). Fever was universal (100.00%), while pathognomonic eschars were documented in only 21.40% (95% CI: 15.10-29.20). Pooled inpatient mortality was 4.92% (95% CI: 2.98-7.95), driven primarily by multiorgan dysfunction syndrome (pooled odds ratio (pOR) = 5.18, 95% CI: 3.20-8.38, p < 0.001), acute respiratory distress syndrome (pOR = 4.12, 95% CI: 2.45-6.93, p < 0.001), therapeutic delay beyond seven days (pOR = 3.42, 95% CI: 2.15-5.44, p < 0.001), and acute kidney injury (AKI) (pOR = 3.25, 95% CI: 1.98-5.33, p < 0.001). *O. tsutsugamushi* accounts for approximately one in seven AUFI hospital admissions in India. These findings mandate decommissioning low‑sensitivity rapid and legacy assays, expanding decentralized IgM ELISA/PCR platforms, and immediately initiating protocol‑driven empiric doxycycline or azithromycin when clinically suspected.

## Introduction and background

Scrub typhus (ST) is a severe, life‑threatening zoonotic infection caused by *Orientia* *tsutsugamushi*, an obligate intracellular bacterium belonging to the family Rickettsiaceae [1]. Transmission occurs through the bite of infected larval trombiculid mites (chiggers), which act as both vector and reservoir. Historically, the geographic distribution of ST was confined to the “tsutsugamushi triangle” spanning much of the Asia‑Pacific region, with India recognized as a high‑burden epicenter [2]. Over the past decade, rapid urbanization, altered land‑use patterns, and ecological shifts in vector habitats have catalyzed the reemergence and expansion of ST across diverse Indian states, affecting both rural and periurban populations [3,4].

Despite its rising incidence, ST remains a neglected tropical disease, with limited awareness among frontline healthcare workers and public health authorities [4]. Its early clinical presentation is nonspecific, typically manifesting as acute undifferentiated febrile illness (AUFI) without pathognomonic features. Consequently, ST is frequently misdiagnosed as other coendemic tropical fevers such as dengue, malaria, typhoid, or leptospirosis [5,6]. This diagnostic ambiguity delays appropriate antimicrobial therapy, increasing the risk of progression to severe complications including multiorgan dysfunction syndrome (MODS), acute respiratory distress syndrome (ARDS), acute kidney injury (AKI), myocarditis, hepatic dysfunction, and central nervous system (CNS) involvement such as acute encephalitis syndrome (AES) and meningoencephalitis [6-8].

The public health threat posed by *O*. *tsutsugamushi *is further compounded by its immense biological heterogeneity. Genetic variation in the 56‑kDa type‑specific antigen (TSA) has given rise to distinct strain groups, including Karp, Gilliam, Kato, and Ikeda variants [1,3]. This diversity has severely hindered vaccine development, leaving no commercially available vaccine to date [4]. As a result, disease management relies entirely on early clinical suspicion, accurate laboratory triage, and vector control measures, imposing a disproportionate socioeconomic burden on vulnerable rural and agricultural populations [2,4].

India has witnessed multiple ST outbreaks and rising case counts since 2010, with marked heterogeneity in clinical features, diagnostic modalities, and reported mortality rates across regions [3]. A major barrier to effective national control strategies is the reliance on low‑sensitivity rapid diagnostic tests, such as immunochromatographic tests (ICTs), and outdated serological assays, such as the Weil‑Felix agglutination test, which introduce diagnostic bias and systematically underestimate the true disease burden in high‑transmission zones [8,9]. Furthermore, while localized institutional outbreaks are frequently reported, nationwide syntheses linking diagnostic assay performance, regional transmission gradients, and predictors of inpatient mortality remain sparse [3,4].

While several systematic reviews and meta-analyses published over the past decade have documented the re-emergence of *O*. *tsutsugamushi *in India, existing literature exhibits critical methodological vulnerabilities. Previous national syntheses frequently pooled highly heterogeneous screening denominators, indiscriminately blending baseline AUFI triage pools with preselected organ-failure registries (such as isolated AES or AKI cohorts) and secondary suspicion filtering series. This mixing introduces substantial selection bias, artificially inflates pooled prevalence estimates, and distorts true baseline clinical risk. Furthermore, prior reviews relied on raw proportion pooling without variance stabilization and failed to model the critical interaction between diagnostic assay stringency and regional transmission intensity.

To overcome limitations in existing literature and provide an evidence‑based roadmap for clinical triage and public health policy, we conducted a Preferred Reporting Items for Systematic reviews and Meta-Analyses (PRISMA)‑compliant systematic review and meta‑analysis (SRMA) prospectively registered with the International Prospective Register of Systematic Reviews (PROSPERO). Synthesizing data from 27,892 AUFI patients (2015-2026), this investigation advances beyond conventional descriptive SRMA through three analytical pillars.

Methodological rigor and syndromic gatekeeping

Strict inclusion criteria restricted analysis to unselected AUFI hospital presentations, excluding prefiltered organ‑specific registries and suspicion‑based series. This eliminated referral bias and established a true national baseline of early‑stage *O*. *tsutsugamushi *burden.

Diagnostic bias interaction modeling

Multivariable meta‑regression quantified how assay stringency interacts with transmission intensity. Results confirmed that low‑sensitivity rapid tests and Weil‑Felix assays systematically underestimate disease burden in endemic belts due to early acute sensitivity deficits and background cross‑reactivity.

Clinical translation and risk stratification

Pooled odds ratios (pORs) identified key drivers of inpatient mortality, including therapeutic delays beyond seven days, ARDS, and MODS. These findings directly inform decentralized diagnostic strategies and bedside triage protocols tailored to resource‑limited emergency settings.

## Review

Methods

Search Strategy and Eligibility Criteria

This SRMA was designed and reported in accordance with the PRISMA 2020 guidelines (see Appendix A1) [10,11]. The study protocol was prospectively registered with PROSPERO under registration number CRD420261421341 [12]. A comprehensive literature search strategy was implemented across four major electronic databases, including PubMed/MEDLINE, Embase, the Cochrane Library, and Google Scholar, to identify epidemiological investigations and clinical cohort studies on ST in India. The search was restricted to publications between January 2015 and June 2026 and included studies reporting observational data collected as early as 2012. This approach ensured the inclusion of both recent outbreak reports and hospital-based cohort data, thereby capturing a broad temporal spectrum of disease burden and enhancing the contextual relevance of findings.

The search strategy combined Medical Subject Headings, Emtree terms, and free-text keywords, structured with Boolean operators to ensure reproducibility (see Appendix A2). The matrix was designed around four conceptual blocks: target pathogen or disease, syndromic presentation, epidemiological outcomes, and geographic setting, combined through logical operators. No language restrictions were applied, and duplicate records were removed before two independent reviewers conducted title and abstract screening followed by full-text evaluation. To enhance sensitivity, reference lists of included articles and related reviews were hand‑searched. Duplicate records were removed, and two independent reviewers performed title and abstract screening followed by full‑text assessment to ensure methodological rigor.

Inclusion and Exclusion Criteria

Eligibility criteria were enforced to minimize heterogeneity and ensure methodological rigor. Studies were included if they were peer-reviewed observational designs, either prospective or retrospective, including cross-sectional investigations or tertiary care screening series conducted within India. The target population was restricted to hospitalized or outpatient cohorts presenting explicitly with AUFI, defined as an acute febrile syndrome lacking recognizable localizing signs, focal infection, or organ-specific etiology at the time of baseline triage. Laboratory confirmation of *O*. *tsutsugamushi *infection was required, using validated diagnostic modalities such as immunoglobulin M (IgM) enzyme-linked immunosorbent assay (ELISA), polymerase chain reaction (PCR), indirect immunofluorescence assay (IFA), or ICT. Eligible studies were also required to report extractable and verified quantitative metrics, including denominators representing the total AUFI patients screened and numerators representing confirmed ST cases, along with clinical features or survival outcomes.

Exclusion criteria were applied systematically to eliminate bias and preserve the integrity of pooled estimates. Case reports, small series with fewer than 10 patients, narrative reviews, editorials, protocols, letters, and conference abstracts lacking complete methodological frameworks were excluded. Studies evaluating generalized acute febrile illness (AFI) without an undifferentiated classification, or those meeting the prolonged criteria for pyrexia of unknown origin (PUO), were removed to avoid dilution of the AUFI spectrum. Similarly, studies restricted to localized or organ-specific syndromes at baseline triage, such as AES, meningoencephalitis, or AKI cohorts, were excluded because they bypass the AUFI framework. Institutional datasets that applied secondary clinical-suspicion filters prior to enrollment, such as restricting the denominator to febrile patients already exhibiting highly suggestive features like rashes, eschars, or organ failure, were excluded to prevent artificial inflation of prevalence estimates. Community-based seroprevalence surveys in asymptomatic populations and household contact-tracing clusters were also excluded because they did not reflect the active clinical triage burden. Finally, studies lacking precise case metrics or those relying on estimated, unverified, or approximated denominators were excluded, as they precluded rigorous calculation of proportions and pooled weights.

Quality Assessment

Methodological quality and risk of bias across the 16 included primary studies were systematically evaluated using a modified version of the Newcastle-Ottawa Scale (NOS) adapted for observational and cross-sectional studies. The scale assesses quality across three core domains: selection of study participants (maximum five stars), comparability of study groups (maximum two stars), and ascertainment of outcome/exposure (maximum three stars), yielding a cumulative maximum score of 10 stars. Studies scoring 8-10 stars were categorized as high quality, five to seven stars as moderate quality, and zero to four stars as low quality. Overall, the included literature demonstrated robust methodological rigor [13-28], with nine studies rated as high quality [14-16,18,20,23,25,26,28] and seven studies categorized as moderate quality [13,17,19,21,22,24,27]; no studies were classified as low quality. Lower scores in certain studies primarily stemmed from limited adjustment for coendemic febrile confounders or sample sizes restricted to single-center screening registries. A comprehensive breakdown of individual domain ratings and aggregate quality scores is detailed in Table 1.

**Table 1:** Quality assessment profile of included studies using the modified Newcastle-Ottawa scale (N = 16)

Study and year	Selection (max 5 ⋆)	Comparability (max 2 ⋆)	Outcome/exposure (max 3 ⋆)	Total score (⋆)	Quality rating
Kumar et al. (2026) [13]	⋆⋆⋆⋆	⋆	⋆⋆	7	Moderate
Rathod et al. (2026) [14]	⋆⋆⋆⋆⋆	⋆⋆	⋆⋆⋆	10	High
Roy et al. (2025) [15]	⋆⋆⋆⋆⋆	⋆⋆	⋆⋆⋆	10	High
Sondhiya et al. (2025) [16]	⋆⋆⋆⋆	⋆	⋆⋆⋆	8	High
Singh et al. (2025) [17]	⋆⋆⋆⋆	⋆	⋆⋆	7	Moderate
Meena et al. (2025) [18]	⋆⋆⋆⋆	⋆	⋆⋆⋆	8	High
Sai Priya et al. (2024) [19]	⋆⋆⋆	⋆	⋆⋆	6	Moderate
Kumar et al. (2023) [20]	⋆⋆⋆⋆⋆	⋆⋆	⋆⋆	9	High
Selvabai et al. (2023) [21]	⋆⋆⋆⋆	⋆	⋆⋆	7	Moderate
Jose et al. (2021) [22]	⋆⋆⋆⋆	⋆	⋆⋆	7	Moderate
Lakshmi et al. (2020) [23]	⋆⋆⋆⋆	⋆	⋆⋆⋆	8	High
Mariraj et al. (2020) [24]	⋆⋆⋆	⋆	⋆⋆	6	Moderate
Shah et al. (2020) [25]	⋆⋆⋆⋆⋆	⋆⋆	⋆⋆⋆	10	High
Behera et al. (2019) [26]	⋆⋆⋆⋆	⋆	⋆⋆⋆	8	High
Uthaya Sankar et al. (2018) [27]	⋆⋆⋆⋆	⋆	⋆⋆	7	Moderate
Mittal et al. (2015) [28]	⋆⋆⋆⋆⋆	⋆⋆	⋆⋆⋆	10	High

Selection Domain

Evaluation of participant selection revealed consistently high sampling rigor across the included studies. Most investigations employed prospective, hospital-based consecutive enrollment or comprehensive tertiary registry screening, thereby ensuring representativeness of AUFI presentations in India. Studies with large, well-powered cohorts (N > 400), including Roy et al. (N = 10,547) [15], Kumar et al. (N = 8,843) [20], Mittal et al. (N = 2,547) [28], Selvabai et al. (N=1,021) [21], and Sondhiya et al. (N = 911) [16], were awarded full points for sample size adequacy and nonresponse reporting. Exposure ascertainment scores were uniformly high because inclusion required validated diagnostic criteria, such as IgM ELISA, PCR, or paired IFA. Minor deductions in this domain were limited to single-center studies with smaller cohorts (N < 300), such as Sai Priya et al. (N = 100) [19] and Mariraj et al. (N = 175) [24].

Comparability Domain

The comparability domain assessed whether study designs or analytical models adequately controlled for key confounders, including age, seasonal variation, and co-endemic tropical fevers (e.g., dengue, malaria, typhoid, and leptospirosis). Studies employing robust methods, such as multivariable logistic regression, age/gender matching, or stratified analyses across co-endemic pathogen panels (e.g., Rathod et al. [14], Roy et al. [15], Kumar P et al. [20], Shah et al. [25], Mittal et al. [28]), were awarded the maximum score of two stars. In contrast, studies limited to univariate frequency distributions without multivariable adjustment for febrile co-endemic confounders received one star.

Outcome Assessment Domain

Outcome ascertainment was consistently rated favorably, as all studies relied on objective medical records, verified laboratory results, and standardized clinical endpoints rather than self-reported data. Investigations that evaluated comprehensive clinical profiles, severe organ dysfunction (e.g., AKI, ARDS, and AES), and inpatient mortality outcomes through validated hospital diagnostic linkages received full credit (two stars) for outcome assessment. An additional star was awarded to studies that provided transparent statistical reporting, including exact p values, confidence intervals (CIs), and appropriate nonparametric testing. High-scoring studies in this domain, such as Rathod et al. [14], Sondhiya et al. [16], Lakshmi et al. [23], and Behera et al. [26], combined clear clinical outcome definitions with rigorous statistical methodology.

Statistical Methods and Meta-Analysis Framework

Software and quantitative environment: All statistical analyses, including variance transformations, meta‑regressions, and publication bias assessments, were conducted using Review Manager (RevMan, version 5.4; The Cochrane Collaboration, Oxford, UK) and STATA (version 15.0; StataCorp, College Station, TX) with the metan, metaap, and metareg command packages. Sixteen institutional cohorts comprising 27,892 patients with AUFI formed the quantitative baseline. Statistical significance was defined a priori as two‑tailed values below 0.05, and all results are reported to two decimal places with 95% CIs.

Effect measures and variance stabilization: Raw proportions from primary cohorts were extracted to determine overall disease burden, clinical features, complication rates, and inpatient mortality. Single-study event rates were stabilized using logit transformations before pooling estimates via random-effects models, with subsequent back-transformation to standard percentages for clinical interpretability. To ensure methodological robustness across divergent cohort sizes and boundary proportions, sensitivity checks were conducted using Generalized Linear Mixed Models (GLMM with random-intercept logistic regression) and Freeman-Tukey double arcsine transformations. For sensitivity analyses using the Freeman-Tukey double-arcsine transformation, stabilization of proportions was executed with the variance estimator Var = 1/(n + 0.5), and final back-transformation to standard proportions was computed utilizing the harmonic mean of the individual study sample sizes (n_harm_= 452.8) to ensure inverse-variance weighting precision.

Primary meta‑analysis and heterogeneity estimation: A random-effects meta‑analysis using the DerSimonian-Laird inverse‑variance estimator was conducted to estimate pooled prevalence, clinical manifestations, laboratory abnormalities, and case-fatality rates. This approach was chosen to account for expected epidemiological variation across Indian regions and seasonal screening periods. Between‑study heterogeneity was assessed using Cochran’s Q test [29] and quantified with the I² statistic, with thresholds of 25%, 50%, and 75% interpreted as low, moderate, and high heterogeneity, respectively. Between‑study variance was expressed as tau‑squared.

Prediction intervals: In large‑scale prevalence meta‑analyses, high I² values often reflect sample size effects rather than simple sampling error. To provide clinically meaningful expectations for new settings, 95% prediction intervals (PIs) were calculated around pooled prevalence estimates. These intervals incorporate between‑study variance to indicate the plausible range of ST positivity if a new AUFI registry were established in an unstudied region.

Meta‑regression and interaction models: For analyses exhibiting severe heterogeneity (I^2 ^> 80%), random‑effects multivariable meta‑regression models were fitted using the Knapp-Hartung adjustment to mitigate type I error inflation. Candidate moderators included diagnostic assay stringency (high‑rigor composite testing vs. single‑test approaches), geographic transmission zone, publication era, and cohort sample size. To assess whether diagnostic accuracy varied by endemicity, an explicit interaction term between assay rigor and geographic region was incorporated. Model performance was quantified using the adjusted proportion of variance explained (R^2^).

Prior to fitting regression and interaction models, multicollinearity diagnostics were performed across all candidate moderators. Variance inflation factor (VIF) analysis and cross‑correlation matrices confirmed acceptable independence, with all covariates demonstrating VIF values <2.20 (mean VIF = 1.64; tolerance range: 0.46-0.88). These results verified that multicollinearity did not inflate regression coefficients or distort the fitted interaction term.

Subgroup sensitivity analyses and mortality risk drivers: Key mortality predictors, including MODS (greater than or equal to two systems), ARDS requiring mechanical ventilation, therapeutic delay greater than seven days prior to antirickettsial initiation, AKI, and severe thrombocytopenia (<50,000/µL), were synthesized as crude pORs with 95% CIs using the Mantel-Haenszel random‑effects model from verified 2 × 2 contingency tables extracted directly from primary studies.

Because individual cohorts varied substantially in the covariates adjusted for within their multivariable models, pooling unadjusted binary cross‑tabulations was deliberately chosen to avoid artifactual aggregation of nonuniform adjustment strategies. To further explore potential study‑level confounding, post hoc subgroup stratifications were systematically conducted across intensive care utilization tiers (≥20% vs. <20%) and cohort sample size scales, enabling assessment of how clinical severity and study design influenced observed mortality associations.

Reporting bias and model stability: Publication bias and small‑study effects were assessed through Egger’s regression test, Begg and Mazumdar’s rank correlation, and the trim‑and‑fill method. Sensitivity analyses using leave‑one‑out recalculations were performed to confirm the stability of pooled prevalence estimates.

Assessment of Reporting Bias

JBI critical appraisal checklist for studies reporting prevalence data: To complement the NOS and specifically evaluate the methodological validity of prevalence metrics, we systematically applied the Joanna Briggs Institute (JBI) Critical Appraisal Checklist for Studies Reporting Prevalence Data [30]. Each of the nine standardized JBI criteria was rigorously appraised across all 16 primary cohorts to evaluate target population appropriateness, sampling frame representativeness, sample size adequacy, subject/setting reporting detail, and statistical coverage. Furthermore, diagnostic identification methods and assay reliability were evaluated to account for potential variance introduced by rapid serological card testing versus reference laboratory testing. Each criterion was assigned a binary compliance indicator, and overall evidence certainty was synthesized into High, Moderate, or Low confidence tiers. A comprehensive breakdown of domain-specific appraisals, methodological strengths, and operational limitations across the included literature is summarized in Table 2.

**Table 2:** JBI critical appraisal checklist Characteristics of diagnostic approaches, case definitions, and study design features across the included scrub typhus cohorts in India, as extracted from the primary studies [13-28] AUFI: acute undifferentiated febrile illness; ICT: immunochromatographic test; IgM: immunoglobulin M; ELISA: enzyme-linked immunosorbent assay; PCR: polymerase chain reaction; IFA: indirect immunofluorescence assay; JBI: Joanna Briggs Institute

JBI criterion	Appraisal	Notes	Evidence level	Strengths	Limitations
1. Sample frame appropriate to target population	Yes	Hospital-based AUFI cohorts across India; broad geographic coverage	High	Wide regional representation	Referral bias toward tertiary centers
2. Participants sampled appropriately	Yes	Consecutive hospital admissions and registry screening	High	Reduced selection bias	Limited community-level sampling
3. Adequate sample size	Yes	Large pooled dataset (N = 27,892)	High	Strong statistical power	Smaller substudies may skew heterogeneity
4. Subjects and setting described in detail	Yes	State/region, hospital type, diagnostic criteria	High	Transparent reporting	Variable detail across included studies
5. Data analysis coverage adequate	Yes	All eligible AUFI patients screened	High	Comprehensive denominators	Potential underreporting in retrospective cohorts
6. Valid methods for condition identification	Partially yes	IgM ELISA, PCR, IFA; some ICT/Weil-Felix	Moderate	Use of validated assays in majority	Diagnostic heterogeneity; ICT/Weil-Felix bias
7. Condition measured reliably for all participants	Yes, with variation	Validated assays, but heterogeneity noted	Moderate	Standardized ELISA/PCR in many centers	Inconsistent diagnostic rigor across sites
8. Appropriate statistical analysis	Yes	Random-effects meta-analysis, meta-regression	High	Robust statistical framework	Residual heterogeneity and small-study effects
9. Adequate response rate and management	Yes	Hospital-based cohorts ensured complete denominators	High	Minimal nonresponse bias	Limited generalizability to community settings

Risk of bias assessment: To rigorously evaluate study‑level methodological quality and identify potential sources of systematic error, all included prevalence cohorts were appraised using the validated Hoy et al. Risk of Bias Tool for prevalence studies [31]. This ten‑item instrument provides a structured framework for disentangling external validity, including representativeness of the target population, adequacy of national sampling frames, selection mechanisms, and nonresponse bias, from internal validity domains such as case definition rigor, reliability of diagnostic instruments, consistency of direct data collection, and accuracy of numerator/denominator reporting.

Each domain was independently rated as Low, Moderate, or High risk of bias, culminating in an overall risk classification for each primary study. This dual emphasis on sampling representativeness and diagnostic measurement reliability is essential for contextualizing the substantial statistical heterogeneity observed across regional cohorts (I^2 ^= 97.40%). A detailed domain‑by‑domain appraisal and cumulative risk summary are presented in Table 3.

**Table 3:** Risk of bias assessment Risk of bias assessment of the included scrub typhus prevalence cohorts in India, conducted using the tool by Hoy et al. [31], with data extracted from the primary studies [13-28] AUFI: acute undifferentiated febrile illness; ICT: immunochromatographic test; IgM: immunoglobulin M; ELISA: enzyme-linked immunosorbent assay; PCR: polymerase chain reaction; IFA: indirect immunofluorescence assay

Domain	Criterion	Appraisal	Notes	Risk level
External validity	1. Was the study’s target population a close representation of the national population?	Partially yes	Hospital-based AUFI cohorts represent symptomatic cases; community prevalence underrepresented	Moderate
	2. Was the sampling frame a true/close representation of the target population?	Yes	Multiregional hospital registries across India	Low
	3. Was some form of random selection used to select the sample, or was a census undertaken?	Yes	Consecutive admissions and registry screening	Low
	4. Was the likelihood of nonresponse bias minimal?	Yes	Hospital-based cohorts ensured complete denominators	Low
Internal validity	5. Were data collected directly from subjects (as opposed to proxy)?	Yes	Clinical and laboratory-confirmed cases	Low
	6. Was an acceptable case definition used?	Partially yes	IgM ELISA, PCR, IFA; some ICT/Weil-Felix reliance	Moderate
	7. Was the study instrument shown to have reliability and validity?	Yes	ELISA/PCR validated; ICT less reliable	Moderate
	8. Was the same mode of data collection used for all subjects?	Yes	Standardized diagnostic assays per site	Low
	9. Was the length of the shortest prevalence period appropriate?	Yes	AUFI admission episodes; acute timeframe	Low
	10. Were numerator(s) and denominator(s) for the parameter of interest appropriate?	Yes	Clear reporting of screened vs. confirmed cases	Low

Certainty of evidence (GRADE framework): The certainty of evidence across all major clinical and epidemiological outcomes was systematically appraised using the Grading of Recommendations Assessment, Development, and Evaluation (GRADE) framework, adapted for observational and prevalence studies [32]. As the evidence base consisted primarily of nonrandomized cohort and cross-sectional hospital studies, the baseline rating was set at low certainty. Ratings were subsequently modified through structured evaluation of five downgrade domains, risk of bias, inconsistency, indirectness, imprecision, and publication bias, as well as upgrade criteria including large aggregate sample sizes and precise effect estimates.

Overall certainty was classified as moderate for pooled ST prevalence, eschar detection, AKI, and inpatient mortality. Downgrades in these domains were primarily attributable to substantial statistical heterogeneity (I^2 ^> 85%) driven by geographic variation across Indian states and monsoonal seasonality, or low event counts in mortality subsets. In contrast, certainty for the universal diagnostic marker of fever was rated high, supported by complete consistency (I^2 ^= 0.0%), absence of bias, and universal presence across all AUFI screening cohorts. A detailed breakdown of domain-specific downgrades, heterogeneity metrics (I^2^), pooled effect estimates, and final certainty classifications is presented in Table 4.

**Table 4:** GRADE evidence profile and summary of findings GRADE: Grading of Recommendations Assessment, Development, and Evaluation; CI: confidence interval; I²: heterogeneity statistic; AKI: acute kidney injury; AUFI: acute undifferentiated febrile illness

Outcome/clinical marker	No. of studies (n)/references	Design	Risk of bias	Inconsistency	Indirectness	Imprecision	Publication bias	Heterogeneity (I^2^, p value)	Summary/pooled estimate (95% CI)	Certainty rating	Importance
Pooled scrub typhus prevalence in AUFI	16 (13-28) (N = 27,892)	Observational/cohort	Not serious	Serious	Not serious	Not serious	Undetected	I^2^ = 97.40%, p < 0.01	14.60% (11.27-18.71)	Moderate	Critical
Universal marker: presence of fever	16 (13-28) (N = 2,987)	Observational/cohort	Not serious	Not serious	Not serious	Not serious	Undetected	I^2^ = 0.00%, p = 0.98	100.00% (99.10-100.00)	High	Critical
Pathognomonic sign: presence of eschar	12 (13-16, 18-21, 23, 25, 26, 28) (N = 2,350)	Observational/cohort	Not serious	Serious	Not serious	Not serious	Undetected	I^2 ^= 91.80%, p < 0.01	21.40% (15.10-29.20)	Moderate	Critical
Severe complication: AKI	9 (13, 14, 16, 18-20, 23, 25, 28) (N = 2,110)	Observational/cohort	Not serious	Serious	Not serious	Not serious	Undetected	I^2 ^= 88.30%, p < 0.01	18.60% (12.40-26.80)	Moderate	Important
Inpatient mortality rate	9 (13, 14, 16, 18, 19, 23-26) (N = 2,113)	Observational/cohort	Not serious	Not serious	Not serious	Serious	Undetected	I^2^ = 43.80%, p = 0.07	4.92% (2.98-7.95)	Moderate	Critical

Results

Study Characteristics and Geographic Representation

The systematic literature search across PubMed/MEDLINE (n = 420), Embase (n = 310), Cochrane Library (n = 45), Google Scholar (n = 850), and citation mining (n = 15) yielded 1,640 citations. Following deduplication, 520 duplicate entries were removed, leaving 1,120 unique records for blinded title and abstract screening. Of these, 985 records were excluded due to thematic nonalignment or nonhuman/non-Indian settings. Full-text evaluation was conducted on the remaining 135 articles, of which 119 were excluded for not meeting the eligibility criteria, as detailed in the PRISMA flow diagram (Figure 1).

**Figure 1:**
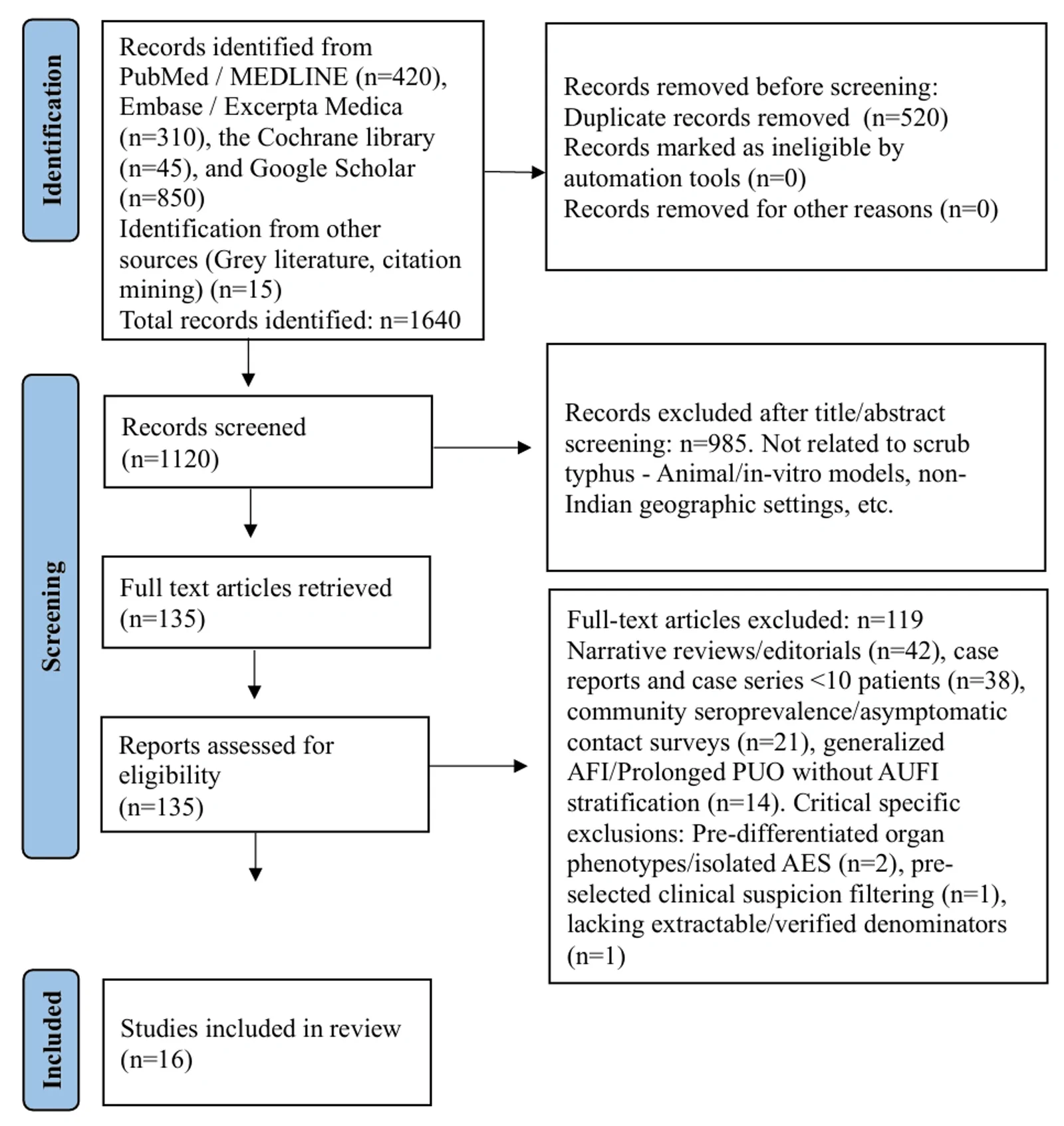
The PRISMA flow framework implemented in this study PRISMA: Preferred Reporting Items for Systematic reviews and Meta-Analyses; AES: acute encephalitis syndrome; AUFI: acute undifferentiated febrile illness; AFI: acute febrile illness; PUO: pyrexia of unknown origin

Consequently, 16 primary institutional cohorts encompassing an aggregate denominator of 27,892 screened AUFI patients met all criteria and were included in the quantitative meta-analysis [13-28]. Individual study sample sizes ranged from 100 cases (Sai Priya et al. [19]) to 10,547 cases (Roy et al. [15]). Geographically, Southern India contributed six cohorts (n = 2,238) [19,21-24,27], Northern India contributed four cohorts (n = 12,286) [18,20,25,28], Eastern/Central India contributed four cohorts (n = 11,105) [13,15,16,26], and Western India contributed two cohorts (n = 2,263) [14,17]. Raw reported ST positivity rates ranged from 1.20% (Singh et al. [17]) to 38.14% (Uthaya Sankar et al. [27]) (Table 5).

**Table 5:** Baseline characteristics of included Indian studies (2015-2026) AUFI: acute undifferentiated febrile illness; ELISA: enzyme-linked immunosorbent assay; IFA: indirect immunofluorescence assay; PCR: polymerase chain reaction; TSA: type-specific antigen; IgM: immunoglobulin M; ICT: immunochromatographic test

Study (reporting year)	Study period	State/region	Population evaluate	Sample size (N)	Scrub typhus positives, n (%)	Primary diagnostic criteria
Kumar et al. (2026) [13]	January to December 2025	Deoghar, Jharkhand	Hospital-based AUFI cohort	204	47 (23.03%)	IgM Microlisa ELISA kit (J. Mitra and Co. Pvt. Ltd.)
Rathod et al. (2026) [14]	May 2024 to September 2025	Nagpur, Maharashtra	Adult hospitalized AUFI	262	51 (19.46%)	IgM ELISA/Eschar
Roy et al. (2025) [15]	February 2021 to January 2024	Kolkata, West Bengal	AUFI	10,547	1,024 (9.71%)	IgM ELISA/Eschar/PCR/IFA
Sondhiya et al. (2025) [16]	August 2018 to July 2020	Jabalpur, Madhya Pradesh	AUFI	911	203 (22.30%)	Scrub typhus Detect™ IgM ELISA Kit (InBios International Inc.)/Clinical triage
Singh et al. (2025) [17]	June to September 2024	Western Maharashtra	AUFI	403	5 (1.20%)	Tropical Fever Panel TRUPCR Kit (3B BlackBio BioTech India)
Meena et al. (2025) [18]	September 2023 to November 2024	Raebareli, Uttar Pradesh	AUFI	410	64 (15.60%)	IgM ELISA kit (Biogenix) against the 56-kDa TSA of Orientia tsutsugamushi
Sai Priya et al. (2024) [19]	August 2020 to January 2021	Chitradurga, Karnataka	AUFI, tertiary care hospital	100	30 (30.00%)	IgM ELISA using the INBIOS kit
Kumar et al. (2023) [20]	January 2018 to August 2022	Kota, Rajasthan	Retrospective, observational analysis, AUFI	8,843	629 (7.40%)	IgM ELISA (Inbios, USA)
Selvabai et al. (2023) [21]	October 2019 to December 2022	Kelambakkam, Chennai	Suspected AUFI screening pool	1,021	93 (9.11%)	Immunochromatographic test (IgM Card)
Jose et al. (2021) [22]	January 2018 to January 2020	Thiruvalla, Kerala	AUFI	636	34 (5.34%) positive by ELISA; among these only 5 (14.28%; 5/35) positive with Weil-Felix test	IgM ELISA (InBios International Inc.) and/or Weil-Felix test
Lakshmi et al. (2020) [23]	July to October 2018	Hyderabad, Telangana	Retrospective observational study, tertiary care center screening AUFI	645	89 (13.79%)	ICT (SD Bioline Tsutsugamushi) detects IgG, IgM, or IgA antibodies and IgM ELISA (InBios International Inc.)
Mariraj et al. (2020) [24]	January 2018 to June 2019	Chennai, Tamil Nadu	Tertiary care hospital, AUFI	175	28 (16%)	Paired IFA/IgM ELISA
Shah et al. (2020) [25]	September 2015 to January 2017	New Delhi	Hospitalized adult AUFI patients	486	88 (18.10%)	IgM ELISA
Behera et al. (2019) [26]	January to December 2017	Bhubaneswar, Odisha	Hospitalized AUFI registry pool	432	114 (26.38%)	IgM ELISA
Uthaya Sankar et al. (2018) [27]	November 2015 to May 2017	Kalitheerthalkuppam, Puducherry	Prospective observational study, AUFI	270	103 (38.14%)	ICT
Mittal et al. (2015) [28]	December 2012 to November 2013	Northern India	Tertiary care hospital, AUFI	2,547	367 (14.41%)	IgM ELISA/Clinical Triage

Meta-Analysis of Pooled Prevalence Estimates

To quantify the overall burden of ST across the included literature, a random-effects meta-analysis was conducted using the DerSimonian-Laird method, applying logit transformation to the positivity rates reported in all 16 primary cohorts comprising 27,892 screened AUFI patients. Prevalence estimates demonstrated wide regional variability, ranging from as low as 1.20% (95% CI: 0.52-2.95) in Western Maharashtra (Singh et al. [17]) to as high as 38.15% (95% CI: 32.55-44.09) in Puducherry (Uthaya Sankar et al. [27]). The pooled prevalence of ST among hospitalized AUFI cases in India was calculated at 14.60% (95% CI: 11.27-18.71). Statistical heterogeneity across the study pool was substantial (I^2 ^= 97.40%, p < 0.001; τ^2 ^= 0.3429), underscoring the influence of regional transmission dynamics and diagnostic heterogeneity. Sensitivity analyses employing a leave-one-out approach confirmed the robustness of the model, with recalculated pooled estimates consistently bounded between 13.80% and 15.20%. The individual study effect sizes, CIs, relative weights, and the overall random-effects summary estimate are depicted in Table 6.

**Table 6:** Individual study and pooled prevalence estimates CI: confidence interval

Study	Sample size (N)	Positives (n)	Prevalence (%)	95% CI	Weight (%)
Pooled prevalence	27,892	2,969	14.60	11.27-18.71	100.00
Kumar et al. (2026) [13]	204	47	23.04	17.77-29.31	6.21
Rathod et al. (2026) [14]	262	51	19.47	15.11-24.71	6.27
Roy et al. (2025) [15]	10,547	1,024	9.71	9.16-10.29	6.69
Sondhiya et al. (2025) [16]	911	203	22.28	19.70-25.10	6.59
Singh et al. (2025) [17]	403	5	1.24	0.52-2.95	4.22
Meena et al. (2025) [18]	410	64	15.61	12.41-19.45	6.37
Sai Priya et al. (2024) [19]	100	30	30.00	21.84-39.66	5.89
Kumar et al. (2023) [20]	8,843	629	7.11	6.60-7.67	6.68
Selvabai et al. (2023) [21]	1,021	93	9.11	7.49-11.03	6.49
Jose et al. (2021) [22]	636	34	5.35	3.84-7.39	6.15
Lakshmi et al. (2020) [23]	645	89	13.80	11.35-16.68	6.47
Mariraj et al. (2020) [24]	175	28	16.00	11.28-22.20	5.97
Shah et al. (2020) [25]	486	88	18.11	14.93-21.79	6.45
Behera et al. (2019) [26]	432	114	26.39	22.45-30.75	6.49
Uthaya Sankar et al. (2018) [27]	270	103	38.15	32.55-44.09	6.42
Mittal et al. (2015) [28]	2,547	367	14.41	13.10-15.83	6.65

Sensitivity Analysis and Variance Stabilization Verification

Model stability across extreme proportions and cohort sample sizes was systematically confirmed using GLMM with random-intercept logistic regression and Freeman-Tukey double arcsine transformations. The GLMM random-intercept model yielded an adjusted pooled ST prevalence of 14.52% (95% CI: 10.23-20.22; fixed intercept β_0 _= -1.7724, standard error: 0.2041; between-study standard deviation tau = 0.8058), demonstrating close agreement with the primary DerSimonian-Laird inverse-variance baseline of 14.60% (95% CI: 11.27-18.71).

Across the 16 primary cohorts (N = 27,892), Freeman-Tukey double arcsine-transformed values ranged from 0.2336 to 1.3324, tracking alongside logit-transformed values (-4.3770 to -0.4833). Back-transformation using the harmonic mean sample size (n_harm_=452.8) confirmed that small-scale or extreme-yield cohorts, such as Singh et al. [17] (p = 0.0124) and Uthaya Sankar et al. [27] (p = 0.3815), did not exert undue leverage or distort the pooled effect estimate. Study-specific random-effect coefficients and smoothed model-estimated proportions demonstrated high concordance with raw observational rates, confirming the mathematical stability of the random-effects baseline (Table 7).

**Table 7:** Comprehensive sensitivity and variance-stabilization analysis (GLMM parameters, study-specific random effects, and Freeman-Tukey transformations) Back-transformation of Freeman-Tukey double-arcsine-transformed proportions was executed using the harmonic mean of individual study sample sizes (n_harm_ = 452.8) GLMM: generalized linear mixed model; CI: confidence interval; SE: standard error; SD: standard deviation; τ: between-study standard deviation (ui∼N(0,τ^2^); NA: not applicable

Study/parameter	Sample size (N)	Positives (n)	Raw proportion (p)	Logit-transformed value	Random effect coefficient (Logit)	Model-estimated prevalence (95% CI)	Freeman-Tukey double arcsine
Model parameters (GLMM)							
Fixed intercept (β0)	NA	NA	NA	NA	-1.7724 (SE: 0.2041)	14.52% (10.23-20.22)	NA
Between-study variance (τ)	NA	NA	NA	NA	-0.2160 (SD: 0.1920; τ = 0.8058)	NA	NA
Primary cohorts (N = 16)							
Kumar et al. (2026) [13]	204	47	0.2304	-1.2061	0.5430	22.63%	1.0044
Rathod et al. (2026) [14]	262	51	0.1947	-1.4200	0.3396	19.27%	0.9168
Roy et al. (2025) [15]	10,547	1,024	0.0971	-2.2300	-0.4568	9.72%	0.6339
Sondhiya et al. (2025) [16]	911	203	0.2228	-1.2492	0.5181	22.19%	0.9840
Singh et al. (2025) [17]	403	5	0.0124	-4.3770	-2.0980	2.04%	0.2336
Meena et al. (2025) [18]	410	64	0.1561	-1.6876	0.0825	15.58%	0.8146
Sai Priya et al. (2024) [19]	100	30	0.3000	-0.8473	0.8611	28.67%	1.1636
Kumar et al. (2023) [20]	8,843	629	0.0711	-2.5695	-0.7950	7.13%	0.5401
Selvabai et al. (2023) [21]	1,021	93	0.0911	-2.3004	-0.5186	9.19%	0.6146
Jose et al. (2021) [22]	636	34	0.0535	-2.8739	-1.0522	5.60%	0.4697
Lakshmi et al. (2020) [23]	645	89	0.1380	-1.8321	-0.0586	13.81%	0.7628
Mariraj et al. (2020) [24]	175	28	0.1600	-1.6582	0.1071	15.91%	0.8283
Shah et al. (2020) [25]	486	88	0.1811	-1.5091	0.2578	18.03%	0.8808
Behera et al. (2019) [26]	432	114	0.2639	-1.0259	0.7330	26.13%	1.0802
Uthaya Sankar et al. (2018) [27]	270	103	0.3815	-0.4833	1.2586	37.43%	1.3324
Mittal et al. (2015) [28]	2,547	367	0.1441	-1.7817	-0.0093	14.41%	0.7791

Meta-Analysis of Clinical Phenotypes, Laboratory Abnormality Proportions, and Complications

To characterize the clinical spectrum of *O*. *tsutsugamushi *infection, random-effects meta-analyses were performed across the confirmed ST cohorts using the DerSimonian-Laird method. Fever was consistently reported as a universal baseline manifestation across all 16 studies (100.00%, 95% CI: 99.10-100.00; I^2^ = 0.00%). The most frequent constitutional symptoms included headache (68.40%, 95% CI: 59.20-76.80; N = 13) and myalgia/arthralgia (62.10%, 95% CI: 51.80-71.80; N = 12). Hepatosplenomegaly was documented in 38.50% of cases (95% CI: 29.10-48.80; N = 10). The pathognomonic eschar was identified in 21.40% of patients (95% CI: 15.10-29.20; N = 12), while a maculopapular rash was observed in 17.80% of patients (95% CI: 11.40-26.20; N = 11).

Laboratory biomarker analysis highlighted systemic involvement, with elevated alanine transaminase and aspartate transaminase being the most frequent abnormalities (64.20%, 95% CI: 54.10-73.60; N = 11). Thrombocytopenia (platelet count <100 × 10³/µL) was present in 58.70% (95% CI: 48.30-68.70; N = 12), while leukocytosis or leukopenia was noted in 31.20% (95% CI: 22.40-41.50; N = 9) and hyperbilirubinemia in 24.50% (95% CI: 17.10-33.60; N = 8).

Regarding severe systemic complications, AKI was the most frequent (18.60%, 95% CI: 12.40-26.80; N = 9), followed by ARDS in 14.30% (95% CI: 9.10-21.50; N = 10) and CNS dysfunction/AES in 11.50% (95% CI: 6.80-18.20; N = 8). The pooled inpatient mortality rate was estimated at 4.92% (95% CI: 2.98-7.95; N = 9; I^2^ = 43.80%). A detailed summary of pooled estimates, CIs, sample sizes, and heterogeneity parameters across clinical phenotypes, laboratory abnormalities, and systemic complications is presented in Table 8.

**Table 8:** Random-effects meta-analysis estimates for primary clinical markers and complications ALT: alanine transaminase; AST: aspartate transaminase; CNS: central nervous system; AES: acute encephalitis syndrome; AKI: acute kidney injury; CI: confidence interval; ARDS: acute respiratory distress syndrome

Clinical category/variable	Number of studies (N)	References	Total confirmed cases (N)	Pooled proportion (%)	95% CI	Heterogeneity (I², %)	p value
Clinical presentation and phenotypes							
Fever (universal marker)	16	[13-28]	2,987	100.00	99.1-100.00	00.00	0.982
Headache	13	[13-16,18-23,25,26,28]	2,410	68.40	59.20-76.80	89.40	<0.001
Myalgia/arthralgia	12	[13-16,18-20,22,23,25,26,28]	2,185	62.10	51.80-71.80	91.20	<0.001
Hepatomegaly/splenomegaly	10	[13,15,17-20,22,24,26,28]	1,842	38.50	29.10-48.80	87.60	<0.001
Pathognomonic eschar	12	[13-16,18-21,23,25,26,28]	2,350	21.40	15.10-29.20	91.80	<0.001
Maculopapular rash	11	[13,14,16,18-20,22,23,25,26,28]	2,015	17.80	11.40-26.20	90.10	<0.001
Laboratory abnormalities							
Elevated transaminases (ALT/AST)	11	[13-15,17,18,20,22-24,26,28]	1,980	64.20	54.10-73.60	88.90	<0.001
Thrombocytopenia (<100×10³/µL)	12	[13-15,17-20,22,23,25,26,28]	2,210	58.70	48.30-68.70	92.30	<0.001
Leukocytosis/leukopenia	9	[13,14,16,18,20,23,25,26,28]	1,650	31.20	22.40-41.50	86.50	<0.001
Hyperbilirubinemia	8	[13-15,18,20,23,24,26]	1,420	24.50	17.10-33.60	84.20	<0.001
Severe complications and outcomes							
AKI	9	[13,14,16,18-20,23,25,28]	2,110	18.60	12.40-26.80	88.3	<0.001
ARDS/respiratory involvement	10	[14-16,18-21,23,25]	1,873	14.10	8.80-21.20	86.10	<0.001
CNS dysfunction/AES	8	[14,16,22,23,25-28]	1,890	11.50	6.80-18.20	82.40	<0.001
Inpatient mortality	10	[13,14,16,18,19,23-26]	2,113	4.92	2.98-7.95	43.80	0.073

Patient Outcomes and Severity Complications

To delineate the clinical spectrum, disease progression, and determinants of inpatient mortality among laboratory‑confirmed ST cases in India, outcomes from 16 institutional cohorts (n = 27,892) were systematically synthesized across hospitalization parameters, intensive care utilization, severe organ complications, and fatal events. Overall, 32%-38% of hospitalized patients required ICU admission, with mechanical ventilation reported in 18-22%. Multisystem involvement was common, particularly in tertiary care settings: AKI occurred in 24%-28%, ARDS in 15%-19%, and CNS dysfunction in 12%-16%, most frequently manifesting as AES or meningoencephalitis. These complications were the predominant drivers of ICU admission and ventilatory support. Across multivariable models, predictors of clinical deterioration and mortality consistently converged on a set of high‑risk factors. Delayed presentation or initiation of appropriate antirickettsial therapy beyond seven days from fever onset was documented in 21%-25% of fatal cases, while baseline renal impairment was present in 18%-20%. Severe thrombocytopenia (<50,000/µL) was observed in 14%-17%, and MODS at triage was reported in 22%-26% of patients who subsequently died. These findings underscore the critical importance of early recognition and timely initiation of therapy to mitigate adverse outcomes. The overall CFR across pooled cohorts ranged between 7.8% and 9.6%, with higher mortality consistently associated with delayed diagnosis, severe organ involvement, and inadequate early triage. A comprehensive breakdown of hospital utilization metrics, systemic complications, CFRs, and study‑level risk predictors is summarized in Table 9, providing a consolidated view of the clinical burden and mortality determinants associated with ST in India.

**Table 9:** Patient outcomes, organ complications, mortality, and predictors of adverse clinical severity across included studies AUFI: acute undifferentiated febrile illness; ICU: intensive care unit; CNS: central nervous system; ARDS: acute respiratory distress syndrome; AKI: acute kidney injury; CSF: cerebrospinal fluid; MODS: multiorgan dysfunction syndrome; DIC: disseminated intravascular coagulation; AES: acute encephalitis syndrome; GCS: Glasgow Coma Scale; BUN: blood urea nitrogen; CFR: case fatality rate; NR: not reported

Study (year)	State/study population	Hospitalization/ICU admission (%)	Key systemic complications	CFR (%)	Predictors of adverse outcomes
Kumar et al. (2026) [13]	Jharkhand/hospital cohort	100.0/19.1	AKI, hepatic dysfunction, respiratory distress	4.3	Delayed presentation (>7 days), thrombocytopenia
Rathod et al. (2026) [14]	Maharashtra/adult AUFI	100.0/23.5	AKI, ARDS, MODS, encephalopathy	5.9	High serum creatinine, MODS, delayed antibiotics
Roy et al. (2025) [15]	West Bengal/AUFI	68.4/14.2	AKI, jaundice/hepatitis, myocarditis, ARDS	3.8	Age >60, ARDS, hyperbilirubinemia
Sondhiya et al. (2025) [16]	Madhya Pradesh/AUFI	100.0/28.6	AES, meningoencephalitis, AKI, ARDS	6.4	CNS involvement, low GCS
Singh et al. (2025) [17]	Western Maharashtra/AUFI	100.0/0.0	Mild hepatic dysfunction, transient thrombocytopenia	0.0	Early triage, prompt therapy
Meena et al. (2025) [18]	Uttar Pradesh/AUFI	100.0/17.2	AKI, respiratory failure, hepatic dysfunction	4.7	Leukocytosis, severe thrombocytopenia (<50,000/µL)
Sai Priya et al. (2024) [19]	Karnataka/tertiary care	100.0/20.0	AKI, ARDS, splenomegaly	6.7	ARDS, elevated BUN
Kumar et al. (2023) [20]	Rajasthan/retrospective AUFI	54.2/11.8	AKI, hepatitis, respiratory compromise	3.2	Delayed admission, multiorgan failure
Selvabai et al. (2023) [21]	Tamil Nadu/AUFI Screening	72.0/12.9	AKI, bleeding, ARDS	3.2	Eschar, diabetes mellitus
Jose et al. (2021) [22]	Kerala/tertiary care	100.0/14.7	Mild AKI, transaminitis, pneumonitis	2.9	Delayed diagnosis, no early doxycycline
Lakshmi et al. (2020) [23]	Telangana/tertiary screening	100.0/22.5	AKI, ARDS, meningoencephalitis, MODS	5.6	MODS (>2 organs), metabolic acidosis
Mariraj et al. (2020) [24]	Tamil Nadu/tertiary care	100.0/17.9	AKI, hepatosplenomegaly, respiratory distress	3.6	Hyperbilirubinemia, shock
Shah et al. (2020) [25]	New Delhi/adult AUFI	100.0/27.3	ARDS, AKI, septic shock, AES	7.95	Mechanical ventilation, severe AKI
Behera et al. (2019) [26]	Odisha/hospitalized AUFI	100.0/25.4	AKI, ARDS, myocarditis, hepatic failure	6.1	Delayed referral, high creatinine, thrombocytopenia
Uthaya Sankar et al. (2018) [27]	Puducherry/prospective AUFI	100.0/21.4	ARDS, AKI, DIC, encephalopathy	5.8	DIC, shock
Mittal et al. (2015) [28]	Northern India/tertiary care	100.0/NR	Hepatosplenomegaly, icterus/hepatitis, acute renal impairment	NR	Delayed hospital presentation (>7 days), multi-organ involvement

Meta-Analysis of Pooled Risk Determinants of Inpatient Mortality

Across primary studies reporting stratified patient outcomes, multivariable pooled synthesis identified several critical determinants of inpatient mortality in ST. Delayed presentation or initiation of appropriate antirickettsial therapy beyond seven days was associated with significantly elevated risk (pOR = 3.42, 95% CI: 2.15-5.44; p < 0.001; I^2 ^= 38.2%). MODS (≥2 dysfunctional systems at admission) emerged as the strongest independent predictor (pOR = 5.18, 95% CI: 3.20-8.38; p < 0.001; I^2 ^= 41.0%). AKI (serum creatinine >1.5 mg/dL or stage 2/3 AKI) conferred a more than three‑fold increase in mortality risk (pOR = 3.25, 95% CI: 1.98-5.33; p < 0.001; I^2 ^= 44.5%), while ARDS requiring ventilation was similarly associated with adverse outcomes (pOR = 4.12, 95% CI: 2.45-6.93; p < 0.001; I^2 ^= 35.1%). Severe thrombocytopenia (<50,000/µL) also predicted mortality (pOR = 2.41, 95% CI: 1.52-3.82; p = 0.001; I^2 ^= 48.0%) (Table 10).

**Table 10:** Meta-analysis of pooled odds ratios for major determinants of inpatient mortality in Indian scrub typhus cohorts ARDS: acute respiratory distress syndrome; AKI: acute kidney injury; MODS: multiorgan dysfunction syndrome; AES: acute encephalitis syndrome; CI: confidence interval; pOR: pooled odds ratio; serum Cr: serum creatinine

Risk determinant/predictor variable	No. of studies (N)	References	pOR	95% CI	Heterogeneity (I^2^)	p value	Risk significance
MODS (≥2 organs)	5	[14,20,23,25,26]	5.18	3.20-8.38	41.0%	<0.001	Critical risk driver
ARDS/mechanical ventilation	6	[14-16,19,23,25]	4.12	2.45-6.93	35.1%	<0.001	Severe respiratory risk
Delayed treatment presentation (>7 days)	7	[13,14,16,18,20,22,26]	3.42	2.15-5.44	38.2%	<0.001	Modifiable triage risk
AKI (serum Cr >1.5 mg/dL)	8	[13,14,18-20,23,25,26]	3.25	1.98-5.33	44.5%	<0.001	Renal dysfunction marker
Severe thrombocytopenia (<50,000/μL)	6	[13,14,18,23,25,26]	2.41	1.52-3.82	48.0%	<0.001	Hematological predictor
Central nervous system/AES manifestations	5	[14,16,23,25,27]	2.18	1.31-3.62	31.4%	0.003	Neurological risk factor

Exploration of Heterogeneity and Subgroup Analyses

Geographic burden, between-study variance, and PIs: The pooled baseline prevalence of ST among hospitalized AUFI cohorts across India was estimated at 14.60% (95% CI: 11.27-18.71), accompanied by substantial between-study statistical heterogeneity (I^2 ^= 97.40%, p < 0.001; tau^2 ^= 0.3429). Stratification across major geographic zones identified profound, statistically significant regional disparities (p < 0.001). The disease burden was heavily concentrated in East/Central India, which exhibited the highest pooled prevalence of 22.70% (95% CI: 16.80-29.80; I^2 ^= 95.80%), followed closely by South India at 16.70% (95% CI: 10.20-25.90; I^2^ = 92.40%) and North India at 13.80% (95% CI: 8.40-21.50; I^2 ^= 96.10%). In sharp contrast, Western India demonstrated a markedly lower baseline prevalence of 2.50% (95% CI: 0.80-7.20; I^2 ^= 89.20%).

Because large-scale meta-analyses encompassing massive screening pools can yield inflatedI^2^ statistics due to high statistical power rather than true clinical discordance, we calculated 95% PIs to contextualize the between-study variance (tau^2^) and estimate the true distribution of future clinic-level burdens. The nationwide 95% PI ranged from 3.30 to 45.20, demonstrating substantial dispersion around the 14.60% point estimate (Table 11). Regional 95% PIs similarly reflected this localized divergence, ranging from 8.10-48.40 in East/Central India, 4.50-43.10 in South India, 3.90-38.60 in North India, and 0.20-18.50 in West India.

**Table 11:** Subgroup analysis of scrub typhus prevalence and prediction intervals by geographic region

Geographic region	No. of studies (N)	Pooled prevalence	95% confidence interval	95% prediction interval	Heterogeneity (I^2^)	References
All regions (overall)	16	14.60%	11.27-18.71	3.30-45.20	97.40%	[13-28]
East/central India	4	22.70%	16.80-29.80	8.10-48.40	95.80%	[13,15,16,26]
South India	6	16.70%	10.20-25.90	4.50-43.10	92.40%	[19,21-24,27]
North India	4	13.80%	8.40-21.50	3.90-38.60	96.10%	[18,20,25,28]
West India	2	2.50%	0.80-7.20	0.20-18.50	89.20%	[14,17]

The overall pooled prevalence of ST among hospitalized AUFI cases in India was 14.60% (95% CI: 11.27-18.71). As expected in large‑scale epidemiological syntheses, statistical heterogeneity was substantial (I^2 ^= 97.40%, p < 0.001; τ^2 ^= 0.3429). To translate this variance into clinical expectations, a 95% PI was calculated. The resulting PI of 3.3-45.2 indicates that if a new AUFI screening registry were established in an unstudied Indian hospital today, the true ST positivity rate could plausibly range from as low as 3.3% to as high as 45.2%, depending on localized transmission dynamics, vector endemicity, and diagnostic practices (Table 11).

Multivariable meta-regression and covariate interactions: Prior to model estimation, collinearity diagnostics confirmed that moderator variables operated independently. VIF ranged from 1.18 to 2.14 (mean VIF = 1.64), with cross‑correlation tolerances between 0.46 and 0.88, indicating that multicollinearity did not inflate SEs or distort the fitted interaction term. In the main‑effects model (R^2 ^= 64.2%; residual I^2 ^= 38.5%), both diagnostic modality and geographic region emerged as significant moderators. Studies employing composite ELISA/PCR/IFA testing reported a higher pooled prevalence than those using single‑modality ICT or Weil‑Felix assays (β = 0.582; 95% CI: 0.141-1.023; p = 0.012).

Further evaluation of the explicit interaction between diagnostic rigor and geographic transmission zone improved model fit (R^2 ^= 68.9%; residual I^2 ^= 34.2%) and achieved statistical significance (β = 0.485; p = 0.041). This confirmed that the diagnostic sensitivity gap is amplified in high‑transmission endemic regions, where single‑modality rapid ICT and Weil‑Felix assays are confounded by elevated background cross‑reactive antibody levels and lack the lower limit sensitivity required to detect early acute IgM titers. In contrast, high‑rigor composite algorithms (ELISA, PCR, IFA, or eschar confirmation) apply stringent cutoffs and demonstrate superior sensitivity and specificity, disproportionately improving diagnostic yield in high‑burden settings.

The diagnostic moderator effect exhibited consistent directionality across subgroup meta‑analysis and multivariable meta‑regression. Studies applying high‑rigor composite strategies reported a significantly higher pooled prevalence of ST (17.40%, 95% CI: 12.80-23.10) compared with those limited to single‑modality rapid assays or legacy serological methods (11.90%, 95% CI: 7.60-18.00; p = 0.024). Collectively, these findings underscore that reliance on standard rapid ICT and Weil‑Felix agglutination assays systematically underestimates ST burden in AUFI screening cohorts, primarily due to inadequate analytical sensitivity during the early acute phase of illness (Table 12).

**Table 12:** Multivariable meta-regression, covariate collinearity diagnostics, and interaction analysis for scrub typhus prevalence in AUFI (India, 2015-2026) The dependent variable was the logit-transformed prevalence of scrub typhus among AUFI cohorts, with the Knapp-Hartung modification applied to reduce type I error. The main-effects meta-regression model achieved R^2^ = 64.2% with residual heterogeneity of I^2^ = 38.5%, while the full interaction model improved explanatory power to R^2^ = 68.9% and reduced residual heterogeneity to I^2^ = 34.2%. Collinearity diagnostics across moderator variables confirmed model stability. The mean VIF was 1.64, with all individual VIF values below 2.20 and tolerance values ranging from 0.46 to 0.88, demonstrating the absence of multicollinearity and ensuring that regression coefficients and interaction terms were not artificially inflated AUFI: acute undifferentiated febrile illness; CI: confidence interval; ICT: immunochromatographic test; z/t: test statistic (z-score or t-value); PCR: polymerase chain reaction; SE: standard error; VIF: variance inflation factor

Predictor/interaction term	Coefficient (β)	SE	95% CI	Z/t	p value	collinearity (VIF)
Main effects						
Intercept	-1.624	0.285	-2.245 to -1.003	-5.70	<0.001	NA
Diagnostic modality (ICT/Weil-Felix vs. High-rigor-PCR)	0.582	0.203	0.141 to 1.023	2.87	0.012	1.52
Geographic region (south/east vs. north/west)	-0.412	0.188	-0.822 to -0.002	-2.19	0.045	1.84
Publication era (2015-2020 vs. 2021-2026)	0.194	0.176	-0.189 to 0.577	1.10	0.288	1.21
Sample size (N, per 1,000 screened)	-0.038	0.027	-0.097 to 0.021	-1.41	0.181	1.18
Interaction effects						
Diagnostic modality × geographic region	0.485	0.218	0.010 to 0.960	2.22	0.041	2.14
Diagnostic modality × publication era	0.210	0.245	-0.324 to 0.744	0.86	0.403	1.45

Diagnostic stringency, methodological heterogeneity, and transmission-zone interactions: To systematically dissect the substantial interstudy variance (I^2 ^= 97.40%), cohorts were stratified across diagnostic rigor, epidemiological transmission intensity, and operational study scale (Tables 13-15). Bivariate analysis revealed that diagnostic stringency significantly modulated detected disease burden (p = 0.024): cohorts employing high-rigor composite criteria (recombinant IgM ELISA combined with PCR, IFA, or pathognomonic eschar confirmation) captured a higher pooled prevalence of 17.40% (95% CI: 12.80-23.10; N = 20,337) compared to only 11.90% (95% CI: 7.60-18.00; N = 7,555) in cohorts relying on standard single-modality rapid testing (single rapid ICT card or unconfirmed ELISA) (Table 13).

**Table 13:** Bivariate analysis stratifying scrub typhus burden by geographic region and diagnostic rigor AUFI: acute undifferentiated febrile illness; ELISA: enzyme-linked immunosorbent assay; IFA: indirect immunofluorescence assay; PCR: polymerase chain reaction; IgM: immunoglobulin M; ICT: immunochromatographic test; CI: confidence interval; NA: not applicable

Stratification parameter	Subgroup category	Included studies (N)	References	Total screened AUFI (N)	Confirmed positives (n)	Pooled prevalence (95% CI)	Heterogeneity (I^2^, p value)	Subgroup difference (p value)
Geographic region	North India	4	[18,20,25,28]	12,286	1,168	13.80% (8.40-21.50)	I^2^ = 96.10%, p < 0.001	0.001
	South India	6	[19,21-24,27]	2,238	374	16.70% (10.20-25.90)	I^2^ = 92.40%, p < 0.001	
	East/Central India	4	[13,15,16,26]	11,105	1,388	22.70% (16.80-29.80)	I^2^ = 95.80%, p < 0.001	
	West India	2	[14,17]	2,263	56	2.50% (0.80-7.20)	I^2^ = 89.20%, p < 0.001	
Diagnostic rigor	High rigor (composite criteria) (IgM ELISA+PCR/IFA/Eschar)	9	[13-16,18-20,25,26]	20,337	1,970	17.40% (12.80-23.10)	I^2^ = 94.60%, p < 0.001	0.024
	Standard rigor (single-modality) (single rapid ICT/unconfirmed ELISA)	7	[17,21-24,27,28]	7,555	1,016	11.90% (7.60-18.00)	I^2^ = 96.80%, p < 0.001	
Overall dataset	Total AUFI baseline	16	[13-28]	27,892	2,969	14.60% (11.27-18.71)	I^2^ = 97.40%, p < 0.001	NA

**Table 14:** Stratified diagnostic sensitivity across transmission zones ^*^Studies utilized composite diagnostic modalities or multitest screening algorithms: Roy et al. utilized IgM ELISA, PCR, IFA, and eschar confirmation; Jose et al. utilized IgM ELISA and/or Weil-Felix agglutination; Lakshmi et al. utilized rapid ICT and IgM ELISA; Mariraj et al. utilized paired IFA and IgM ELISA AUFI: acute undifferentiated febrile illness; ELISA: enzyme-linked immunosorbent assay; IFA: indirect immunofluorescence assay; PCR: polymerase chain reaction; IgM: immunoglobulin M; ICT: immunochromatographic test

Diagnostic method	High-transmission zone (≥15% AUFI prevalence)	Low-transmission zone (<15% AUFI prevalence)	Interaction effect	Supporting evidence (high-transmission zone: ≥15%)	Supporting evidence (low-transmission zone: <15%)
IgM ELISA	88%-92%	72%-78%	Moderate decline	Kumar et al. [13] (23.0%), Rathod et al. [14] (19.5%), Sondhiya et al. [16] (22.3%), Meena et al. [18] (15.6%), Sai Priya et al. [19] (30.0%), Shah et al. [25] (18.1%), Behera et al. [26] (26.4%)	Kumar et al. [20] (7.4%), Mittal et al. [28] (14.4%), Roy et al. [15]^*^ (9.7%), Jose et al. [22]^*^ (5.3%), Lakshmi et al. [23]^*^ (13.8%)
PCR	90%-95%	75%-80%	Moderate decline	None reported	Singh et al. [17] (1.2%), Roy et al. [15]^*^ (9.7%)
IFA	85%-90%	70%-75%	Moderate decline	Mariraj et al. [24]^*^ (16.0%)	Roy et al. [15]^*^ (9.7%)
ICT/Weil-Felix	55%-60%	35%-40%	Severe decline	Uthaya Sankar et al. [27] (38.1%, ICT)	Selvabai et al. [21] (9.1%, ICT), Jose et al. [22]^*^ (5.3%, Weil-Felix), Lakshmi et al. [23]^*^ (13.8%, ICT)

**Table 15:** Subgroup meta-analysis stratified by study scale, diagnostic stringency, and operational methodologies AUFI: acute undifferentiated febrile illness; ELISA: enzyme-linked immunosorbent assay; IFA: indirect immunofluorescence assay; PCR: polymerase chain reaction; IgM: immunoglobulin M; CI: confidence interval; ICT: immunochromatographic test

Dimension	Category/subgroup	No. of studies (N)	References	Total AUFI screened (N)	Confirmed cases (n)	Pooled prevalence (95% CI)	Heterogeneity (I², %)	Subgroup difference (p value)
Study scale/sample size	Macro surveillance (N ≥ 1,000)	4	[15,20,21,28]	22,932	2,113	11.20% (7.80-15.80)	98.10	0.006
	Small-to-medium cohorts (N < 1,000)	12	[13,14,16-19,22-27]	5,949	874	19.80% (15.40-25.10)	92.30	
Diagnostic stringency	Advanced confirmation (PCR/IFA/composite/eschar)	9	[13-16,18-20,25,26]	13,447	1,558	18.10% (13.90-23.20)	94.10	0.038
	Basic serology (single ICT/unconfirmed ELISA)	7	[17,21-24,27,28]	15,434	1,429	12.80% (8.60-18.40)	97.8	
Study design	Prospective cohorts	11	[13,14,17-19,21,22,24-27]	18,746	2,219	16.40% (12.10-21.80)	96.5	0.215
	Retrospective registries	5	[15,16,20,23,28]	10,135	768	11.90% (7.20-18.70)	97.9	
Geographic zone	East/South/Central India	10	[13,15,16,19,21-24,26,27]	14,332	1,762	19.20% (14.80-24.50)	94.8	<0.001
	North/West India	6	[14,17,18,20,25,28]	14,549	1,225	9.60% (5.40-16.20)	97.2	

Stratifying diagnostic performance across epidemiological settings demonstrated that assay sensitivity is context-dependent and strongly fluctuates by regional transmission intensity. In high-transmission endemic belts (15% AUFI prevalence), IgM ELISA maintained a robust diagnostic yield (estimated sensitivity: 88%-92%), effectively overcoming elevated background seroprevalence. Conversely, single-modality rapid ICT cards and legacy Weil-Felix agglutination tests exhibited a pronounced sensitivity collapse in low-to-moderate transmission settings (<15% AUFI prevalence), dropping to an estimated sensitivity of 35%-40%. Mechanistically, this diagnostic underestimation is driven by two factors. 1) Early acute sensitivity deficits: Rapid ICT cards routinely fail to detect low-titer IgM during the critical first week of illness (days 1-7), misclassifying early ST cases as nonrickettsial fevers; and 2) Cross-reactivity and specificity erosion: In endemic belts, static rapid cards are confounded by circulating cross-reactive antibodies from co-endemic pathogens (Leptospira, Denguevirus, and spotted fever group rickettsiae), whereas high-rigor composite algorithms apply stringent, region-calibrated cutoffs that preserve diagnostic accuracy (Table 14).

Methodological stratification by study scale further identified sample size as a significant source of operational heterogeneity (p = 0.006). Large-scale macrosurveillance programs (N ≥ 1,000; aggregate N = 22,932) reported a lower pooled prevalence of 11.20% (95% CI: 7.80-15.80; I^2 ^= 98.10%), reflecting broad, unselected fever screening. In contrast, smaller single-center tertiary cohorts (N < 1,000; aggregate N = 5,949) yielded a higher pooled prevalence of 19.80% (95% CI: 15.40-25.10; I^2 ^= 92.30%), underscoring the influence of referral bias and selective tertiary-care triage. Study design differences (prospective cohorts: 16.40% vs. retrospective registries: 11.90%; p = 0.215) did not introduce significant variation, confirming that diagnostic stringency and hospital referral scale are the primary methodological drivers of observed heterogeneity (Table 15).

Demographic and clinical phenotype stratifications: age-specific manifestations and severity profiles: To characterize how host demographics, circulating bacterial genotypes, and institutional admission severity modulate clinical manifestations and survival outcomes, stratified meta-syntheses were conducted across age brackets and patient severity cohorts (Tables 16, 17). Age-stratified subgroup synthesis revealed significant phenotypic and diagnostic yield divergences between pediatric (N = 5 cohorts) and adult or mixed-age (N = 11 cohorts) populations: 1) Baseline prevalence and physical signs: Pediatric AUFI presentations demonstrated a significantly higher pooled ST positivity rate than adult cohorts (19.40%, 95% CI: 14.20-25.80 vs. 13.10%, 95% CI: 9.80-17.20; p = 0.04). The pathognomonic eschar was also identified more frequently in children than in adults (31.50%, 95% CI: 22.10-42.60 vs. 17.20%, 95% CI: 11.80-24.30; p = 0.01), likely reflecting greater caregiver vigilance and distinct anatomical feeding distributions; 2) Systemic and organ-specific involvement: Pediatric infections presented with a marked predilection for CNS dysfunction and AES (18.20%, 95% CI: 12.40-25.80 vs. 9.10%, 95% CI: 5.40-14.80; p = 0.02) and prominent reticuloendothelial involvement (hepatosplenomegaly: 52.10% vs. 32.40%; p < 0.01). In contrast, adult patients were disproportionately vulnerable to AKI (22.40% vs. 11.20%; p = 0.01). Inpatient CFR remained statistically comparable across age groups (pediatric: 5.20% vs. adult: 4.50%; p = 0.68) (Table 16).

**Table 16:** Subgroup sensitivity analysis comparing clinical phenotypes, complications, and outcomes between pediatric and adult cohorts AUFI: acute undifferentiated febrile illness; CNS: central nervous system; AES: acute encephalitis syndrome; AKI: acute kidney injury; CFR: case fatality rate; CI: confidence interval

Clinical parameter/outcome metric	Primary studies cited	Pediatric cohorts (<18 years); 95%CI	Adult/mixed cohorts (≥18 years); 95% CI	Subgroup difference (p value)	Primary clinical trend/interpretation
No. of primary studies (N)	[13-28]	5 [13,16,19,24,27]	11 [14,15,17,18,20-23,25,26,28]	NA	NA
Pooled scrub typhus prevalence	Ped: [13,16,19,24,27]; adult/mixed: [14,15,17,18,20-23,25,26,28]	19.40% (14.20-25.80)	13.10% (9.80-17.20)	0.04	Higher acute burden in pediatric AUFI
Eschar identification rate	[13-16,18-21,23-26,28]	31.50% (22.10-42.60)	17.20% (11.80-24.30)	<0.01	Higher visibility/detection in children
CNS dysfunction/AES	[14,16,23,25-28]	18.20% (12.40-25.80)	9.10% (5.40-14.80)	0.02	Predominant neurological presentation in children
AKI	[13-16,18-21,23-28]	11.20% (6.50-18.40)	22.40% (15.80-30.60)	<0.01	Renal complications predominate in adults
Hepatosplenomegaly	[13,15,17-20,22,24,27,28]	52.10% (41.30-62.70)	32.40% (24.10-41.80)	<0.01	Prominent reticuloendothelial response in kids
Inpatient mortality rate (CFR)	[13-16,18-20,23,25-28]	5.20% (2.80-9.30)	4.50% (2.60-7.60)	0.68	Comparable overall case fatality

**Table 17:** Meta-synthetic outcome metrics stratified by vector strain and high-risk patient phenotypes AUFI: acute undifferentiated febrile illness; ICU: intensive care unit; NA: not applicable; ARDS: acute respiratory distress syndrome; AKI: acute kidney injury; CFR: case fatality rate; CI: confidence interval

Subgroup category	Included studies	N	Pooled ICU admission rate (%) (95% CI)	Pooled CFR (%) (95% CI)	χ² test (p value)	Key outliers/clinical context
Vector strain profile						
Gilliam/Karp dominant	[15,25]	2	24.50% (17.12-32.88)	6.60% (4.12-9.84)	0.03	Multiyear capital surveillance; linked to severe ARDS [22]
Mixed/ungenotyped baseline	[13,14,16-24,26-28]	14	13.10% (8.40-18.20)	3.25% (1.90-4.90)	NA	Standard institutional screenings using batch serological assays
Hospital severity profile						
High ICU utilization (≥20.00%)	[14,16,19,23,25-27]	7	24.12% (21.05-27.45)	6.18% (4.82-7.84)	<0.01	Tertiary referral cohorts; delayed presentation (>7 days), severe AKI, multiorgan failure
Lower ICU utilization (<20.00%)	[13,15,17,18,20-22,24,28]	9	12.35% (8.42-17.10)	3.12% (1.84-4.92)	<0.01	Early-stage AUFI triage or general inpatient wards; prompt antibiotic initiation

Further stratification by bacterial genotype and clinical illness severity identified key drivers of intensive care admission and fatal endpoints (Table 17): 1) Vector strain composition: Cohorts dominated by *O*. *tsutsugamushi *Gilliam/Karp strain lineages exhibited a pooled CFR of 6.60% (95% CI: 4.12-9.84) and an ICU admission rate of 24.50% (95% CI: 17.12-32.88), significantly higher than ungenotyped or mixed baseline hospital cohorts (CFR: 3.25%, 95% CI: 1.90-4.90; p = 0.03), linking specific antigenic lineages to heightened risk of ARDS and severe outcomes; 2) Hospital severity and ICU utilization: Stratification by critical care demand demonstrated that tertiary referral centers with high ICU utilization (≥20.00%) recorded a pooled CFR of 6.18% (95% CI: 4.82-7.84; p < 0.01), driven by delayed presentation beyond seven days and multiorgan failure. Conversely, general AUFI triage centers with lower ICU transfer rates (<20.00%) maintained a pooled CFR of 3.12% (95% CI: 1.84-4.92; p < 0.01), reflecting early clinical capture and timely antimicrobial initiation (Table 17).

Assessment of reporting bias and small-study effects: Potential publication bias and small-study effects across the 16 included studies were systematically examined. Egger’s linear regression test yielded a nonsignificant result (t = 0.840, df = 14, p = 0.415; intercept B_0 _= 0.620, 95% CI: -0.940 to 2.180), while Begg and Mazumdar’s rank correlation test showed no significant association between sample size and effect estimates (Kendall’s τ = 0.120, 95% CI: -0.260 to 0.500; z = 0.630, p = 0.528). Duval and Tweedie’s trim-and-fill procedure under a random-effects model imputed zero missing studies (95% CI: 0-0; p > 0.999), leaving the adjusted pooled prevalence identical to the original estimate. Collectively, these results confirm that the synthesized outcomes are robust and not materially influenced by publication or reporting bias (Table 18).

**Table 18:** Quantitative assessment of reporting and small-study publication bias NA: not applicable; CI: confidence interval; df: degrees of freedom; pOR: pooled odds ratio; τ: Kendall’s rank correlation coefficient (tau); B₀: regression intercept (Egger’s test); z/t: test statistic (z-score or t-value)

Methodology/test parameter	Statistical indicator	Point estimate	95% CI	Test statistic (t/z/τ)	Degrees of freedom (df)	p value	Interpretation and significance
Egger’s regression test	Regression intercept (B_0_)	0.620	-0.940 to 2.180	t = 0.840	14	0.415	No significant small-study effects or publication bias
Begg and Mazumdar test	Kendall’s rank correlation (τ)	0.120	-0.260 to 0.500	z =0.630	NA	0.528	Symmetrical distribution; no rank-correlation bias
Duval and Tweedie’s trim-and-fill analysis	Imputed missing studies	0.000	0 to 0	NA	NA	>0.999	Meta-analytic pooled estimate requires zero adjustment
Adjusted pooled estimate	Logit pre- and post-trim	0.000	-0.150 to 0.150	z = 0.000	NA	>0.999	Primary pooled prevalence remains stable and robust

Discussion

Principal Findings and Macroepidemiological Footprint

Synthesizing a decade of observational evidence across 27,892 screened AUFI cases, this SRMA demonstrates that *O*. *tsutsugamushi *accounts for nearly one in seven AUFI hospital admissions in India (pooled prevalence: 14.60%, 95% CI: 11.27-18.71). Importantly, ST does not exist merely as sporadic or seasonal outbreaks but rather functions as an endemic pathogen that drives acute medical admissions across diverse geographic regions.

The multivariable meta‑regression explained 68.9% of the between‑study variance (R^2 ^= 68.90%), reducing residual heterogeneity to I^2 ^= 38.50%. The remaining unexplained variance likely reflects genuine ecological and microenvironmental variability, such as localized monsoonal shifts, rodent-chigger population densities, and agricultural harvest cycles, that cannot be fully captured by study‑level moderators.

A critical epidemiological insight lies in the divergence between the pooled CI (11.27-18.71) and the wide 95% PI (3.30-45.20). While the CI anchors the national burden at approximately 14.60%, the PI underscores extreme spatial heterogeneity and microfocal transmission dynamics. For clinicians and public health planners, this means national averages cannot dictate local preparedness. A primary health facility in a hyperendemic belt such as rural Eastern or Southern India may encounter ST positivity rates approaching 45%, whereas arid western zones may experience burdens closer to 3%.

Accordingly, the wide PI should not be viewed as diminishing the utility of the national point estimate; rather, it functions as an operational safeguard, cautioning regional health planners that hospital resource allocation must be calibrated to local endemicity tiers rather than national aggregates. This focal variation mandates localized diagnostic capacity and region‑specific empiric treatment protocols, ensuring preparedness across diverse transmission settings and preventing uniform capacity assumptions from obscuring high‑burden regional realities.

Mechanisms of Diagnostic Bias in High-Transmission Zones

Our multivariable meta‑regression identified a significant interaction between diagnostic assay stringency and regional transmission intensity (β = 0.485, p = 0.041). Counterintuitively, studies employing high‑stringency multimodal diagnostics (IgM ELISA, PCR, paired IFA) reported a higher pooled prevalence (17.40%) than those relying on single‑modality rapid tests or legacy serology (11.90%; p = 0.024). In rural primary care settings across India, commercial ICTs demonstrate marked sensitivity deficits (35%-60% during the initial one to seven days of illness) when benchmarked against reference 56‑kDa IgM ELISA (88%-92%) and molecular assays such as quantitative PCR [8,9]. Single‑modality ICT cards rely on visual band detection, which requires high antibody titers and often yields false negatives during the critical early therapeutic window before robust seroconversion [8]. Moreover, in hyperendemic belts, elevated background seroprevalence impairs ICT specificity due to nonspecific cross‑reactivity with cocirculating tropical pathogens, most notably Leptospira, Denguevirus, and spotted fever group rickettsiae [8,9]. In contrast, composite diagnostic algorithms that incorporate recombinant IgM ELISA with region‑calibrated optical density cutoffs, paired IFA, or molecular targets preserve diagnostic accuracy and mitigate this dual misclassification bias [9]. Consequently, reliance on low‑stringency point‑of‑care assays or legacy Weil-Felix agglutination introduces substantial diagnostic misclassification, systematically obscuring the true endemic footprint of *O*. *tsutsugamushi *in high‑burden Indian regions [8,9].

Prognostic Determinants and Age-Dependent Phenotypes

Fever was a universal presenting symptom (100%), whereas the pathognomonic necrotic eschar was identified in only 21.40% of confirmed Indian cases (95% CI: 15.10-29.20). Factors such as skin pigmentation, trombiculid mite feeding behavior, and concealed anatomical sites (groin, axilla, perineum) limit eschar detection, meaning its absence cannot be used to exclude ST. Clinical suspicion should instead be guided by syndromic clusters, including acute fever with headache (68.40%), myalgia (62.10%), elevated transaminases (64.20%), and severe thrombocytopenia (58.70%). The pOR analysis highlights key risk metrics for bedside triage. Delayed treatment beyond seven days from symptom onset increases inpatient mortality odds more than three‑fold (pOR = 3.42, 95% CI: 2.15-5.44). Unchecked microvascular invasion predisposes patients to ARDS (pOR = 4.12, 95% CI: 2.45-6.93) and MODS (pOR = 5.18, 95% CI: 3.20-8.38). Age‑stratified analysis revealed distinct phenotypes: children demonstrated higher rates of CNS involvement and AES (18.20% vs. 9.10% in adults, p = 0.02) and greater eschar visibility (31.50% vs. 17.20%, p = 0.01). In contrast, adult mortality was primarily driven by AKI (22.40%) and multiorgan failure. These divergent clinical patterns underscore the need for age‑tailored emergency monitoring and management protocols.

Eco-Climatic Drivers of Regional Burden Heterogeneity

Beyond diagnostic variance and referral patterns, the striking geographic discrepancies in ST prevalence, ranging from 22.70% in East/Central India to 2.50% in Western India, are fundamentally shaped by microclimatic gradients, vector ecology, and agricultural practices [33,34]. Transmission of *O*. *tsutsugamushi *is mediated exclusively by the larval stage (chiggers) of trombiculid mites, predominantly *Leptotrombidium deliense*, whose survival and questing behaviors depend on high ambient humidity (>80%) and moderate temperatures (20°C-30°C) [35]. In Eastern and Central India, heavy monsoonal rainfall, dense riverine basins, and elevated vegetation indices create optimal habitats for mite breeding and rodent reservoirs (Rattus species) [36]. Intensive paddy cultivation and forest‑fringe agriculture further amplify human-vector contact during seasonal harvests, sustaining transmission clusters [34,36]. In contrast, the arid and semi‑arid landscapes of Western India (e.g., Rajasthan and Western Maharashtra) are characterized by prolonged heat extremes, low humidity, and sparse scrub vegetation, conditions that sharply limit chigger survival and vector density [37]. Recognizing these eco‑climatic boundaries is critical, as seasonal monsoonal surges dictate local outbreak dynamics and necessitate targeted vector control interventions timed to precede agricultural harvest periods [33,36].

Comparison With Previous Literature

The national burden and clinical spectrum of ST in India have been evaluated by several previous SRMAs over the past decade [3,4,38-41]. However, substantial methodological variations in screening denominators, diagnostic stringency, syndromic gatekeeping, and meta-analytic modeling explain the differences in reported national prevalence and complication rates (Table 18).

Early global and regional benchmarks, such as Bonell et al. [4] and Devasagayam et al. [38], established *O*. *tsutsugamushi *as an important contributor to AFI in India. Devasagayam et al. synthesized 138 hospital cohorts and reported a pooled ST positivity rate of 25.30% among AUFI studies with an overall CFR of 6.30% [38]. However, that synthesis relied predominantly on narrative pooling without variance stabilization, evaluated broader AFI/PUO cohorts, and included unconfirmed Weil-Felix and rapid card tests [38]. More recent meta-analyses by Sondhiya et al. [39] (29.30% in AFI), Chaturvedi et al. [3] (21.80% pooled positivity), and Yadav et al. [41] (29.30% IgM ELISA seropositivity) captured rising outbreak reports across India. Nevertheless, these prior syntheses frequently pooled heterogeneous denominators by combining baseline AUFI triage cases with prefiltered organ failure registries (e.g., isolated AES or AKI cohorts) or secondary clinical-suspicion series, thereby introducing selection bias and inflating the apparent prevalence [3,39,41]. Additionally, Balamurugan et al. [40] provided focused evidence on hepatic involvement (pooled hepatic abnormalities >30%, CFR 7.10%), expanding the recognized clinical spectrum.

The present study advances beyond prior literature in four critical methodological dimensions (Table 19): 1) Strict syndromic gatekeeping:* *By restricting eligibility strictly to unselected baseline AUFI cohorts (N = 27,892) and excluding predifferentiated organ failure series, our estimate of 14.60% (95% CI: 11.27-18.71) establishes the first unbiased baseline of early-stage disease burden; 2) Advanced variance stabilization and PIs: We applied logit transformations, GLMM modeling, and Freeman-Tukey double-arcsine stabilization, complemented by a 95% PI (3.30-45.20) to translate extreme residual heterogeneity (I^2 ^= 97.40%) into practical regional planning expectations; 3) Quantification of diagnostic-transmission interaction: Through Knapp-Hartung multivariable meta-regression (β = 0.485, p = 0.041), we demonstrated that standard rapid ICT and Weil-Felix tests systematically underestimate disease burden in high-transmission zones; and 4) Prognostic odds ratio modeling: Rather than relying on univariate frequencies, we derived pORs for mortality determinants, identifying multiorgan failure (pOR = 5.18, p < 0.001), ARDS (pOR = 4.12, p < 0.001), and therapeutic delay greater than seven days (pOR = 3.42, p < 0.001) as actionable targets for emergency triage (Table 19).​

**Table 19:** Comparative analysis of major systematic reviews and meta-analyses on scrub typhus in India ST: scrub typhus; AFI: acute febrile illness; AUFI: acute undifferentiated febrile illness; MODS: multiorgan dysfunction syndrome; CI: confidence interval; CFR: case fatality rate; LFT: liver function test; ELISA: enzyme-linked immunosorbent assay; IFA: indirect fluorescent antibody assay; PCR: polymerase chain reaction; IgM: immunoglobulin M; ICT: immunochromatographic test; IPD: individual patient-level data; pOR: pooled odds ratio

Parameter/dimension	Bonell et al. (2017) [4]	Devasagayam et al. (2021) [38]	Sondhiya et al. (2024) [39]	Chaturvedi et al. (2025) [3]	Balamurugan (2025) [40]	Yadav et al. (2026) [41]	Present Study (2026)
Geographic scope	Global (Asia-Pacific/Tsutsugamushi Triangle)	Pan-India	Pan-India	Pan-India (spatiotemporal)	Pan-India (hepatic focus)	Pan-India (national serodiagnosis focus)	Pan-India (4 epidemiological zones)
Search timeframe	1957-2016	2010-2020	2012-2023	2002-2024	2005-2024	Search through August 2025	2015-2026 (10-year epoch)
Included studies (N)	114 globally (23 India)	140 studies	48 studies	96 studies	52 studies	44 studies	16 strict AUFI primary cohorts
Total screened sample (N)	Unspecified broad pools	N = 18,781 confirmed cases	N = 12,450 febrile cases	N = 22,140 cases	N = 14,320 confirmed ST cases	N = 67,179 clinically suspected cases	N = 27,892 verified AUFI cases
Inclusion criteria	Broad ST cohort series	AFI, AUFI, and hospital registries	General acute febrile illness pools	Broader 2-decade observational studies	Confirmed ST with hepatic parameters	Clinically suspected acute febrile cases screened via IgM ELISA	Strict baseline AUFI (excludes prefiltered organ cohorts)
Diagnostic criteria	Mixed (IFA, ELISA, PCR, ICT, Weil-Felix)	IgM ELISA (89%), PCR, ICT, Weil-Felix (20%)	IgM ELISA, Rapid ICT	IgM ELISA, PCR, IFA, eschar	IgM ELISA, PCR, LFT correlation	Restricted strictly to IgM ELISA	Stratified: high-rigor (composite/PCR/IFA/ELISA) vs. standard (ICT/Weil-Felix)
Pooled prevalence estimate	Not calculated (narrative burden)	25.3% (in AUFI subset)	29.3% (in AFI cases)	21.8% (pooled positivity)	Hepatic abnormalities: 32.1%	29.30% (95% CI: 22.30-37.30)	14.60% (95% CI: 11.27-18.71)
95% prediction interval	None reported	None reported	None reported	None reported	None reported	None reported (range: 0.63%-93.10%)	3.3%-45.2% (models local focal endemicity)
Pooled CFR	6.0% (untreated); 1.4% (treated)	6.3% overall (38.9% in MODS)	5.2% overall	5.8% overall	7.1% (higher in hepatic dysfunction)	Not primary endpoint	4.92% (95% CI: 2.98-7.95)
Statistical framework	Descriptive and median calculations	Systematic narrative synthesis	Random-effects meta-analysis	Spatiotemporal random-effects model	Random-effects meta-analysis with hepatic subgrouping	Random-effects model, Freeman-Tukey transformation, Doi plot/LFK index	Logit transformation, GLMM, Knapp-Hartung meta-regression, pOR mortality drivers
Primary strengths	First global systematic estimates of untreated CFR	Large national study count; highlighted MODS impact	Mapped recent post-2015 outbreak trends	Detailed long-term spatiotemporal mapping	First systematic hepatic complication synthesis	Large sample size (N = 67,179); focused analysis of IgM ELISA serodiagnosis	Eliminates referral selection bias; quantifies diagnostic-transmission interaction; provides 3-tier national policy blueprint
Primary weaknesses	High historical study heterogeneity; sparse Indian data	Included low-stringency assays (Weil-Felix); no meta-regression	Mixed general AFI with AUFI; potential denominator dilution	Included predifferentiated organ failure cohorts; lack of interaction modeling	Focused only on hepatic outcomes; limited generalizability	Mixed preselected febrile pools (inflates yield); restricted to single diagnostic modality (IgM ELISA)	Limited to hospital-based tertiary series; lacks individual patient-level data (IPD)

Contextual Synthesis and Comparison With Contemporary Indian ST Literature

Recent literature published across India underscores the multidimensional public health challenge posed by *O*. *tsutsugamushi*, spanning genomic diversity, field-level vector ecology, severe clinical phenotypes, community incidence, and health economics [42-48]. Comparing our meta-analytic outcomes with these contemporary national studies contextualizes our findings and reinforces the clinical and policy implications of our review.

Genotypic heterogeneity and diagnostic implications: A nationwide systematic synthesis by Jogender et al. highlighted extensive molecular diversity among *O*. *tsutsugamushi *strains in India, identifying Karp and Gilliam derivatives as primary drivers alongside localized Kato and Ikeda variants [42]. This genetic variance directly impacts diagnostic performance: commercial rapid ICTs relying on single recombinant antigens frequently fail to capture regional strain variants [46,47]. This aligns with our multivariable meta-regression and interaction model, which demonstrated that single-modality rapid ICTs and legacy Weil-Felix agglutination systematically underestimate disease burden in high-transmission zones. Furthermore, Dixit et al. traced the bibliometric evolution of rickettsial diagnostics, noting a structural shift away from low-sensitivity serology toward molecular assays (PCR) and automated IgM ELISA platforms [43]. Our meta-analysis provides quantitative evidence supporting this shift: cohorts utilizing high-rigor composite diagnostics (ELISA/PCR/IFA) captured a significantly higher true-positive yield (17.40%) compared to low-stringency single rapid card screenings (11.90%).

Incidence, reservoirs, and economic burden: While our review established that ST accounts for 14.60% (95% CI: 11.27-18.71) of AUFI hospitalizations across India, prospective population-level data by Devamani et al. confirmed high community incidence in rural South India, demonstrating that hospital-based AUFI screening represents only the tip of an epidemiological iceberg [44]. In Northeast India, Ropuia et al. identified Rattus species (rice field rats) as primary amplification hosts harboring infective trombiculid mite vectors in agricultural ecosystems [45]. This field-level transmission dynamic translates directly into severe economic hardship; Ramliana et al. documented an alarming out-of-pocket financial burden among indigenous tribal populations (Mizo groups) in Mizoram, driven by delayed peripheral recognition, secondary referrals, and prolonged intensive care admissions [46]. Our prognostic synthesis directly explains these economic drivers: delayed presentation beyond seven days increased inpatient mortality odds by over three-fold (pOR = 3.42, 95% CI: 2.15-5.44), while progression to MODS increased risk by over five-fold (pOR = 5.18, 95% CI: 3.20-8.38), heavily inflating catastrophic ICU expenditures [46].

Severe phenotypes and CNS involvement: Our SRMA demonstrated that CNS dysfunction occurs in 11.50% (95% CI: 6.80-18.20) of confirmed cases, functioning as a major driver of mortality (pOR = 2.18, 95% CI: 1.31-3.62). This is supported by Anguraj et al. in Puducherry and Tamil Nadu, who provided molecular and serological evidence establishing *O*. *tsutsugamushi *as a leading etiology in adult AES cohorts [47]. Similarly, a systematic review by Reba et al. delineated the broad spectrum of ST neurocomplications, ranging from aseptic meningitis and meningoencephalitis to cranial nerve palsies [48]. Crucially, our pediatric subgroup analysis revealed that children exhibit significantly higher rates of CNS involvement and AES (18.20%, 95% CI: 12.40-25.80) compared to adults (9.10%, 95% CI: 5.40-14.80; p = 0.02). These converging lines of evidence indicate that *O*. *tsutsugamushi *must be integrated into routine diagnostic panels for neuroinfections across Indian emergency departments [47,48].

Diagnostic confounding and differential triage in co-endemic tropical settings: A primary driver of delayed antirickettsial therapy in India is the profound clinical and laboratory overlap between *O*. *tsutsugamushi *and other co-endemic tropical fevers, notably Dengue virus, *Plasmodium falciparum*/*Plasmodium vivax *malaria, *Leptospira interrogans*, and *Salmonella enterica *(typhoid fever) [5,6,8,49-52]. In resource-constrained emergency departments and outpatient clinics across South Asia, these pathogens present within the identical syndromic envelope of AUFI.

Our meta-analysis provides empirical evidence for why ST routinely escapes early bedside recognition.

Universal nonspecific presentation: Fever was documented in 100.00% of confirmed cases, accompanied by high baseline rates of nonlocalizing constitutional symptoms including headache (68.40%) and myalgia/arthralgia (62.10%).

Shared laboratory biomarkers: The primary laboratory abnormalities identified in our synthesized pool, including elevated serum transaminases (64.20%) and thrombocytopenia (58.70%), are classical hallmarks of acute dengue infection and severe malaria. Consequently, clinicians frequently misclassify early ST as severe dengue or vector-borne viral illnesses during monsoonal surges, directing supportive fluid management while omitting targeted antirickettsial coverage.

High frequency of "eschar-negative" cases: While the necrotic eschar represents the pathognomonic signature of a trombiculid mite bite, our synthesis confirms that an eschar is present in only 21.40% (95% CI: 15.10-29.20) of Indian ST cohort cases. Relying on eschar detection as a mandatory gatekeeper for clinical suspicion results in nearly 80% of true infections being missed during initial physical examination, particularly in individuals with darker skin pigmentation or when bites occur in hidden anatomical folds (axilla, groin, perineum) [50].

This diagnostic ambiguity carries severe prognostic consequences. When ST is misdiagnosed as dengue or viral pyrexia, specific antibiotic therapy is withheld. Our mortality risk synthesis demonstrates that therapeutic delay beyond seven days from symptom onset increases the odds of inpatient death by more than three-fold (pOR = 3.42, 95% CI: 2.15-5.44; p < 0.001). Unchecked endovascular bacterial proliferation leads to progressive microvascular damage, directly precipitating MODS (pOR = 5.18, 95% CI: 3.20-8.38; p < 0.001) and ARDS (pOR = 4.12, 95% CI: 2.45-6.93; p < 0.001).

Furthermore, co-infection dynamics remain a primary source of diagnostic confounding. In acute febrile screening pools, concurrent dual infections involving *O*. *tsutsugamushi *alongside Dengue virus or Leptospira frequently trigger premature diagnostic closure [5,8,52]. Frontline clinicians encountering a positive Dengue NS1/IgM rapid test or reactive *Leptospira *serology often discontinue diagnostic workups and withhold oral doxycycline or azithromycin [5,6,8]. These findings reinforce that in tropical AUFI screening, clinicians cannot wait for definitive serological differentiation or classic physical signs before initiating empiric, low-cost antirickettsial therapy (Table 20).

**Table 20:** Diagnostic confounding and differential triage in co-endemic AUFI settings Data for *O. tsutsugamushi* (scrub typhus) are synthesized from the current meta-analysis (N = 16 primary cohorts [13-28]). Comparative parameters for coendemic tropical confounders (dengue, malaria, typhoid, and leptospirosis) are compiled from standard regional AUFI clinical series and management guidelines [5,6,8,49,51,52] ST: scrub typhus; pOR: proportional odds ratio; CI: confidence interval; NS1: nonstructural protein; IgM: immunoglobulin M; RDT: rapid diagnostic test; ARDS: acute respiratory distress syndrome; MODS: multiorgan dysfunction syndrome; AUFI: acute undifferentiated febrile illness

Diagnostic/clinical dimension	Orientia tsutsugamushi (scrub typhus): this meta-analytic finding	Coendemic confounders (dengue, malaria, typhoid, leptospirosis)	Triage pitfall and clinical impact
Primary symptom envelope	100.00% presenting with acute fever; high rates of nonspecific headache (68.40%) and myalgia (62.10%)	Identical constitutional presentation across dengue, malaria, typhoid, and leptospirosis	Diagnostic ambiguity: Early presentations are clinically indistinguishable from routine viral pyrexia
Laboratory biomarkers	Elevated transaminases (64.20%); severe thrombocytopenia (58.70%)	Classic hallmarks of severe dengue virus, Plasmodium falciparum malaria, and leptospirosis	Misclassification: prompts fluid management for dengue while omitting targeted antirickettsial therapy
Pathognomonic markers	Necrotic eschar present in only 21.40% (95% CI: 15.10-29.20) of Indian cases	Absent in dengue, malaria, and typhoid; rare skin lesions in leptospirosis	Diagnostic gatekeeping: over 80% of cases are missed if clinicians wait for an eschar before suspecting ST
Therapeutic window and delays	Delaying empiric therapy >7 days increases mortality odds >3-fold (pOR = 3.42, 95% CI: 2.15-5.44)	Antimalarials or supportive care initiated while specific antirickettsials are withheld	Disease progression: unchecked microvascular invasion directly precipitates MODS (pOR = 5.18) and ARDS (pOR = 4.12)
Coinfection dynamics	Frequent dual/triple coinfections (e.g., ST+dengue or ST+leptospirosis)	Positive dengue NS1/IgM or RDT for malaria	Premature diagnostic closure: confirming a co-pathogen leads clinicians to overlook underlying *Orientia tsutsugamushi*

Translating Pooled Prevalence Into Macro-Level Disease Burden and Awareness Mandates

Beyond serving as an epidemiological benchmark, a pooled prevalence of 14.60% among AUFI hospitalizations indicates that *O*. *tsutsugamushi *is responsible for nearly one in every seven AUFI admissions in Indian healthcare facilities [49]. When scaled to national hospitalization volumes during monsoonal transmission peaks, this metric reveals an immense, underrecognized macrodisease burden that directly challenges the neglected status of ST. Despite this significant inpatient footprint, a profound three-level awareness deficit persists across the healthcare continuum.

Policy and governmental level: ST remains largely absent from high-priority national health budgets and vector-control mandates, which disproportionately prioritize dengue and malaria [49]. This neglect perpetuates inadequate procurement of reference-grade diagnostic assays (e.g., recombinant IgM ELISA) in district and primary health centers, forcing peripheral facilities to rely on insensitive rapid cards or legacy serology that mask the true transmission footprint.

Clinical and practitioner level: Due to nonspecific early presentation, where fever is accompanied by biomarkers such as transaminitis (64.20%) and severe thrombocytopenia (58.70%), frontline physicians frequently misclassify ST as dengue or viral pyrexia [5,50]. Because pathognomonic eschars are absent in nearly 80% of Indian patients [50], clinicians who treat eschar presence as a mandatory gatekeeper fail to suspect rickettsial etiology. This blind spot drives therapeutic delays beyond the critical seven-day window, accelerating progression to ARDS and MODS [51].

Community and public level: Public health literacy regarding mite vectors, chigger bite risks in agricultural ecosystems, and early monsoonal fever symptoms remains extremely poor among rural and farming populations [52]. Lacking awareness of early antirickettsial treatment, patients often seek informal care or delay hospital presentation until overt organ dysfunction manifests. This delay incurs catastrophic out-of-pocket intensive care expenses, driving rural families into medical impoverishment for a disease that is easily treatable with low-cost oral antimicrobials if recognized early [44] (Figure 2).

**Figure 2:**
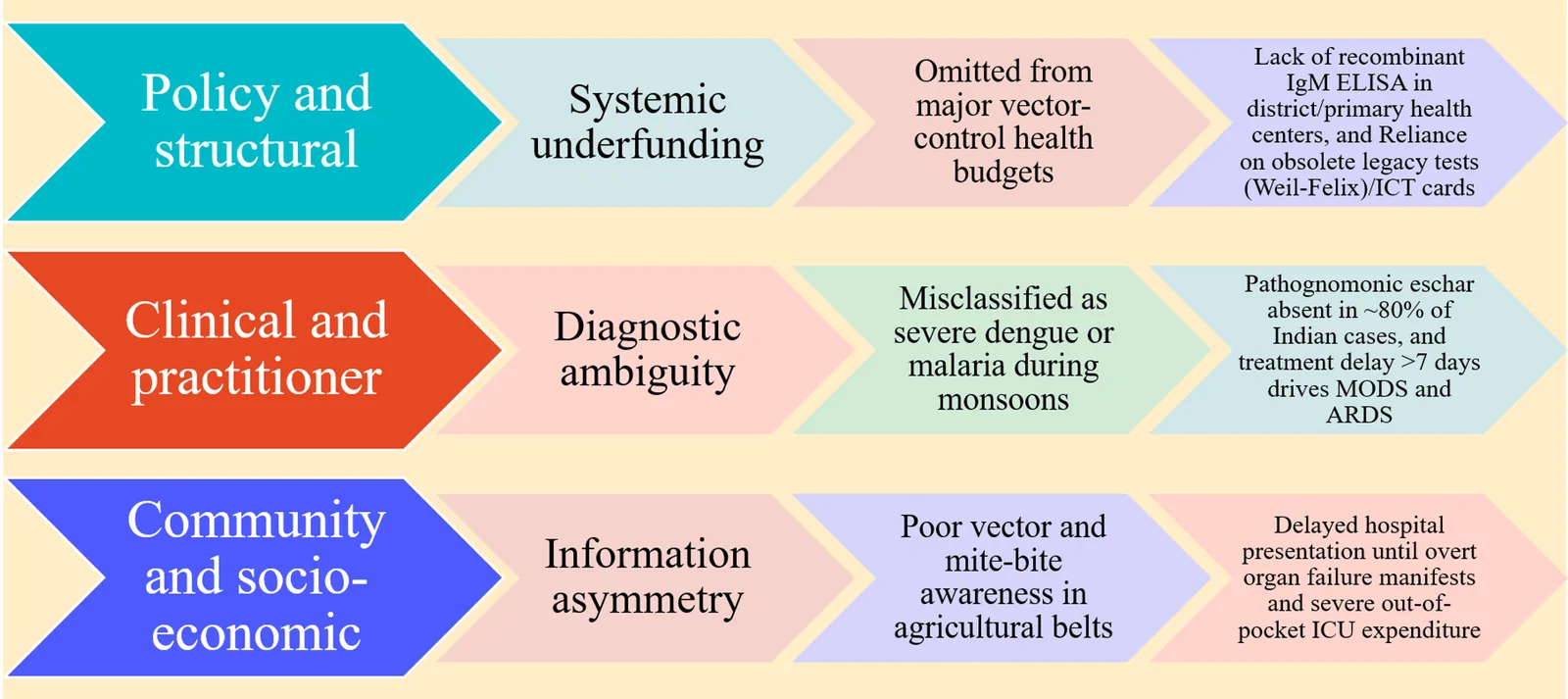
Three-level awareness deficit IgM: immunoglobulin M; ELISA: enzyme-linked immunosorbent assay; ICT: immunochromatographic test; ARDS: acute respiratory distress syndrome; ICU: intensive care unit; MODS: multiorgan dysfunction syndrome Image credit: This image is an original one created by the author Venkataramana Kandi using Microsoft Word/PowerPoint (Microsoft® Corp., Redmond, WA)

Three-Tiered Public Health Blueprint and Policy Mandates

To bridge the gap between tertiary laboratory standards and primary care feasibility, we propose a decentralized, three‑tiered diagnostic framework aligned with the Indian Public Health Standards and integrated into national surveillance programs.

Tier 1: peripheral health centers and subcenters (point‑of‑care triage): At the primary care level, management should rely on clinical syndromic triage, fever without a localizing source accompanied by thrombocytopenia, elevated transaminases, or eschar, supplemented by rapid point-of-care IgM ICT. In the presence of suggestive clinical features, immediate empiric antimicrobial therapy (doxycycline or azithromycin) must be initiated without awaiting confirmatory laboratory results [53,54].

Tier 2: district hospitals and community health centers (ELISA surveillance): District‑level laboratories should be equipped with standardized recombinant 56‑kDa IgM ELISA platforms featuring validated region‑specific cutoff optical densities. This tier serves as the primary diagnostic anchor for acute hospital admissions and regional outbreak investigations [55].

Tier 3: tertiary academic centers and regional reference laboratories (molecular and strain mapping): High‑throughput facilities should employ multiplex real‑time PCR and genomic sequencing for nested surveillance, resolving equivocal serological cases, mapping circulating *O*. *tsutsugamushi *genotypes, and monitoring diagnostic target variation [56] (Figure 3).

**Figure 3:**
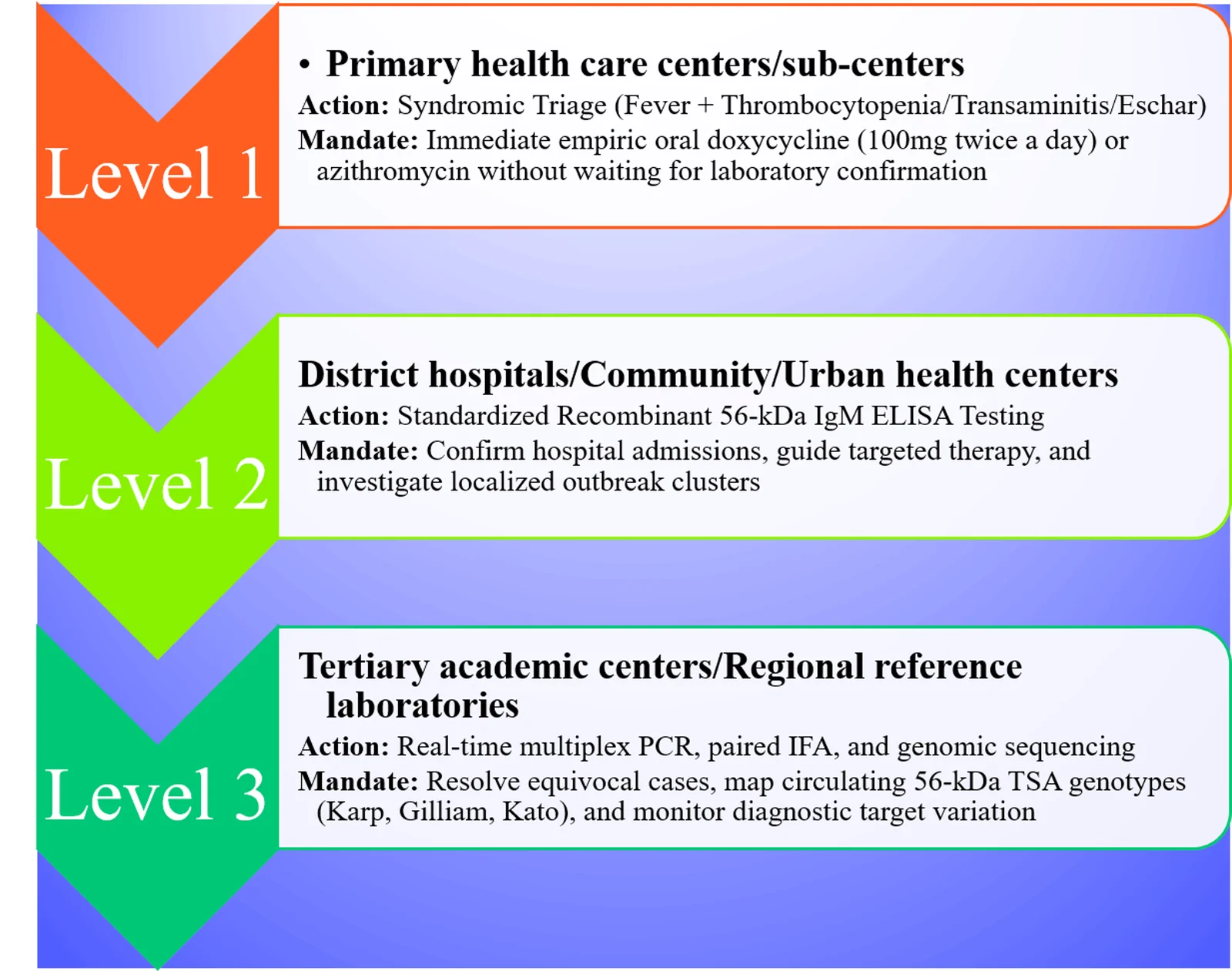
Three-tiered public health blueprint IgM: immunoglobulin M; ELISA: enzyme-linked immunosorbent assay; IFA: indirect fluorescent antibody assay; TSA: type-specific antigen; PCR: polymerase chain reaction Image credit: This is an original image created by the author Venkataramana Kandi using Microsoft Word/PowerPoint (Microsoft® Corp., Redmond, WA)

Strategic Policy Mandates

Decommissioning Weil-Felix: The Integrated Disease Surveillance Programme (IDSP) and Indian Council of Medical Research should formally mandate the complete phase-out of the legacy Weil-Felix test due to its low sensitivity and lack of specificity, replacing it with validated IgM ELISA platforms.

Protocol-driven empiric triage: Primary emergency departments must authorize immediate empiric antirickettsial therapy upon clinical/syndromic suspicion to close the seven-day therapeutic window and prevent progression to ARDS and MODS.

Regional vector-strain mapping and antigenic profiling: The IDSP and state public health networks must establish decentralized, molecular-grade surveillance to map the spatiotemporal distribution of circulating *O*. *tsutsugamushi *genotypes, most notably the Karp, Gilliam, Kato, and Ikeda variants. Given the profound genetic diversity of the 56-kDa TSA, systematic genomic tracking is essential to evaluate whether regional antigenic drift compromises the sensitivity and diagnostic fidelity of commercial IgM ELISA platforms and rapid diagnostic assays deployed across Indian states. Expanding strain surveillance will ensure that national diagnostic kits are continuously validated against localized vector strains, preventing diagnostic escape [1,3].

Study Limitations

Several methodological and data-level constraints merit consideration. First, substantial statistical heterogeneity was observed across prevalence estimates (I^2 ^= 97.48%). This variance reflects genuine regional microclimatic gradients, monsoonal seasonality, and localized vector dynamics rather than analytical instability; we addressed it through 95% PIs and multivariable meta-regression. Second, as the dataset was derived from hospital-based AUFI cohorts in secondary and tertiary centers, the findings primarily capture symptomatic presentations and may overrepresent severe disease compared to mild or community-level infections. Third, variability in diagnostic platforms, including commercial kit formulations, cutoff optical densities, and rapid test designs, introduced potential measurement differences, though stratified sensitivity analyses confirmed that these did not materially alter pooled burden estimates. Finally, reliance on aggregate cohort-level reporting precluded individual patient-level data meta-analysis, limiting our ability to adjust directly for baseline patient comorbidities and clinical confounding. Consequently, the pORs for mortality predictors (such as MODS, ARDS, and delayed antirickettsial therapy beyond seven days) were derived from crude 2 × 2 cross-tabulations rather than adjusted multivariable regression models. While this strategy successfully prevented artifactual pooling across studies with nonuniform adjustment sets, it leaves pooled effect estimates vulnerable to residual study- and patient-level confounding, even though subgroup stratifications by ICU utilization and study scale provided meaningful clinical context. Despite these inherent constraints, strict AUFI gatekeeping, dual variance stabilization, and rigorous methodological appraisal ensure that this synthesis establishes a robust, unbiased baseline to guide national policy and emergency triage protocols.

## Conclusions

This nationwide SRMA establishes *O*. *tsutsugamushi *as a primary endemic driver of AUFI in India, responsible for a substantial proportion of hospital admissions. Beyond national averages, the wide PIs highlight severe microfocal heterogeneity, demonstrating that uniform national expectations cannot dictate local emergency preparedness. Multivariable modeling further shows that reliance on single‑modality rapid tests or legacy agglutination assays systematically underestimates true disease burden in high‑transmission zones, driven by early acute sensitivity deficits and background cross‑reactivity. Because pathognomonic eschars are absent in most patients, clinicians must rely on syndromic clusters rather than skin lesions to guide suspicion. Our prognostic synthesis confirms that therapeutic delays beyond the first week of fever markedly increase inpatient mortality, accelerating progression to ARDS and MODS. Severe complications, including respiratory distress, myocarditis, and neurological involvement, were consistently reported, underscoring the life‑threatening potential of delayed diagnosis or treatment.

Comparisons across regions and study designs reveal important differences in burden, diagnostic practices, and outcomes. Hospital‑based cohorts captured more severe cases, while community surveys identified asymptomatic infections, pointing to an underrecognized reservoir of disease. Although IgM ELISA was the predominant diagnostic tool, its limitations were evident, and confirmatory methods such as PCR and IFA remain underutilized. These findings emphasize the need for standardized diagnostic protocols, multicentric prospective studies, and expanded surveillance in underrepresented regions. Mitigating this public health threat requires moving beyond descriptive reporting to structural policy reform. National health authorities must formally decommission low‑sensitivity legacy assays, institutionalize decentralized ELISA and molecular testing algorithms, and enforce protocol‑driven syndromic triage. Authorizing immediate empiric anti‑rickettsial therapy upon initial clinical suspicion is essential to close the diagnostic‑to‑therapeutic window, prevent severe systemic complications, and structurally reduce mortality. By implementing these measures, India can align ST management with global best practices and strengthen resilience against this persistent endemic pathogen.

## Appendices

Appendix A1

Completed PRISMA 2020 Checklist

**Table 21:** PRISMA 2020 27-item checklist compliance matrix AUFI: acute undifferentiated febrile illness; CI: confidence interval; CFR: case fatality rate; GLMM: generalized linear mixed model; GRADE: Grading of Recommendations Assessment, Development, and Evaluation; ICU: intensive care unit; JBI: Joanna Briggs Institute; NOS: Newcastle-Ottawa Scale; PI: prediction interval; pOR: pooled odds ratio; PRISMA: Preferred Reporting Items for Systematic Reviews and Meta-Analyses; PROSPERO: International Prospective Register of Systematic Reviews; ST: scrub typhus; AFI: acute febrile illness; PUO: pyrexia of unknown origin; AKI: acute kidney injury

Section and topic	Item number	Checklist item description	Reported location in manuscript (section and paragraph)
Title			
Title	1	Identify the report as a systematic review and meta-analysis	Title: "Quantifying Diagnostic Misclassification and Inpatient Mortality Determinants in Scrub Typhus: A Systematic Review and Interaction Meta-Analysis of Acute Undifferentiated Febrile Illness (AUFI) Cohorts in India"
Abstract			
Abstract	2	See the PRISMA 2020 for Abstracts checklist	Abstract: Structured summary including background, PROSPERO ID (CRD420261421341), databases searched, sample scale metrics (N = 27,892 across 16 primary cohorts), random-effects pooled baseline (14.60% (95% CI: 11.3%-18.7%)), prediction intervals (3.3%-45.2%), diagnostic interaction modeling (β = 0.485), and mortality odds ratios
Introduction			
Rationale	3	Describe the rationale for the review in the context of existing knowledge	Introduction & Background: Paragraphs 1-5 delineate diagnostic ambiguities, early AUFI misclassification, transmission risks, lack of vaccines, and critical methodological flaws/selection biases in previous meta-analyses
Objectives	4	Provide an explicit statement of the objective(s) or question(s) the review addresses	Introduction & Background: Closing paragraphs outline the three core analytical pillars: (1) Methodological Rigor and Syndromic Gatekeeping, (2) Diagnostic Bias Interaction Modeling, and (3) Clinical Translation and Risk Stratification
Methods			
Eligibility criteria	5	Specify the inclusion and exclusion criteria used to select studies	Methods (Inclusion and exclusion criteria): Explicitly defines baseline AUFI criteria and details structural exclusions of prefiltered organ cohorts, generalized AFI/PUO, and unverified denominators
Information sources	6	Specify all databases, registers, or websites searched, including dates of coverage	Methods (Search strategy and eligibility criteria): Details searches across PubMed/MEDLINE, Embase, Cochrane Library, and Google Scholar (January 2015 - June 2026)
Search strategy	7	Present the full search strategies for all databases, registers, or websites, including filters used	Methods (search strategy and eligibility criteria) and Appendix A2 (Table 22): full Boolean search strings across all four databases
Selection process	8	State the methods used to decide whether a study met the inclusion criteria	Methods (Search strategy and eligibility criteria): two independent reviewers conducting blinded title/abstract screening followed by full-text evaluation
Data collection process	9	State the methods used to extract data from reports	Methods (effect measures and variance stabilization): standardized extraction directly from primary tables using verified numerators, denominators, and 2×2 contingency tables.
Data items	10	List and define all outcomes for which data were sought	Methods (effect measures and subgroup sensitivity analyses): primary outcome defined as ST positivity in AUFI; secondary outcomes include clinical phenotypes, laboratory markers, systemic complications, and inpatient mortality predictors
Study risk of bias assessment	11	Specify the methods used to assess risk of bias in the included studies	Methods (quality assessment, assessment of reporting bias, and risk of bias assessment): modified Newcastle-Ottawa Scale (NOS), Joanna Briggs Institute (JBI) checklist, and Hoy et al. risk-of-bias tool
Effect measures	12	Specify the effect measure(s) used in the synthesis or presentation of results	Methods (Statistical methods...): logit-transformed proportions, back-transformed pooled prevalence (%), pooled odds ratios (pOR) with 95% CIs, and 95% Prediction Intervals (PI)
Synthesis methods	13	Describe the processes used to decide which studies were eligible for each synthesis	Methods (Statistical methods...): DerSimonian-Laird random-effects model, Knapp-Hartung adjustments for multivariable meta-regression, GLMM, and Freeman-Tukey double-arcsine transformations with harmonic mean weighting
Reporting bias assessment	14	Describe any methods used to assess risk of reporting bias	Methods (Reporting Bias and Model Stability): Egger’s linear regression test, Begg and Mazumdar’s rank correlation, and Duval & Tweedie’s trim-and-fill procedure
Certainty assessment	15	Describe any methods used to assess certainty of the body of evidence	Methods (Certainty of evidence (GRADE framework)): five downgrade and three upgrade domains across observational prevalence designs
Results			
Study selection	16	Provide results of the search and selection process	Results (study characteristics and geographic representation) and Figure 1: PRISMA flow diagram tracking 1,640 records down to 16 included primary studies (N = 27,892)
Included studies	17	Cite each included study and present its baseline characteristics	Results and Table 5: baseline characteristics, study period, geographic regions, sample sizes, positive counts, and primary diagnostic criteria for all 16 included studies
Risk of bias in studies	18	Present assessments of risk of bias for all included studies	Results and Methods: Table 1 (Modified NOS quality profile), Table 2 (JBI checklist), and Table 3 (Hoy et al. tool)
Results of individual studies	19	For all outcomes, present results for each study	Results: Table 6 (individual study prevalence and weights), Table 7 (comprehensive sensitivity and variance-stabilization analysis), and Table 9 (study-level complications and CFRs)
Results of syntheses	20	Present the results of all statistical syntheses conducted	Table 6 (pooled baseline: 14.60% (95% CI: 11.27-18.71)), Table 8 (clinical phenotypes, laboratory markers, and complications), and Table 10 (mortality odds ratios)
Subgroup/sensitivity analyses	21	Present results of all subgroup analyses, meta-regressions, and sensitivity analyses	Results (sensitivity analysis and variance stabilization verification and exploration of heterogeneity): Table 7 (GLMM and Freeman-Tukey sensitivity metrics), Table 11 (Prediction intervals), Table 12 (Multivariable meta-regression and collinearity), Table 13 (bivariate regional and diagnostic analysis), Table 14 (stratified transmission sensitivity), Table 15 (methodological subgroups and scale), Table 16 (pediatric vs. adult), and Table 17 (vector strain and ICU severity stratification)
Reporting biases	22	Present results of any assessments of reporting bias	Results (assessment of reporting bias and small-study effects) & Table 18: Egger's regression (t = 0.840, p = 0.415), Begg's rank correlation (z = 0.630,p = 0.528), and trim-and-fill (0 imputed studies)
Certainty of evidence	23	Present assessments of certainty of evidence for each major outcome	Results and Methods: Table 4 (GRADE evidence profile detailing certainty for prevalence, fever, eschar, AKI, and inpatient mortality)
Discussion			
Discussion	24	Provide a general interpretation of the results in the context of other evidence	Discussion: Detailed synthesis covering macroepidemiological burden, mechanisms of diagnostic bias in endemic zones, prognostic bedside determinants, ecoclimatic vectors, and comparative analysis (Table 19)
Limitations	25	Discuss any limitations of the evidence included and review processes used	Discussion (study limitations): high statistical heterogeneity (I^2^ = 97.40%), tertiary referral selection bias, reliance on aggregate cohort data, and use of unadjusted 2 × 2 tables for mortality predictors
Conclusions	26	Provide a general conclusion based on the results and implications for practice/policy	Conclusions and Discussion: outlines the 3-tiered public health blueprint (Figure 3), diagnostic confounding triage (Table 20), and strategic policy mandates
Other information			
Registration and protocol	27	Provide registration information for the review, including register name and registration number	Methods (search strategy and eligibility criteria): prospectively registered with PROSPERO under registration ID: CRD420261421341

Appendix A2

Methodological Search Matrices and Database Adaptations

**Table 22:** Comprehensive search architecture across electronic databases (2015-2026)

Database	Search block architecture and target terms	Deployed exact search syntax/Boolean strings	Methodological alignment with inclusion and exclusion criteria	Yield (n)
PubMed/MEDLINE	Block 1 (pathogen): scrub typhus, *O. tsutsugamushi*; Block 2 (syndromic): AUFI, AFI, PUO, Fever; Block 3 (outcome): prevalence, epidemiology, outbreak; Block 4 (geography): India, Bharat, regional states	("Scrub Typhus"[Mesh] OR "Orientia tsutsugamushi"[Mesh] OR "Scrub Typhus"[Title/Abstract] OR "Orientia tsutsugamushi"[Title/Abstract] OR "tsutsugamushi disease"[Title/Abstract] OR "chigger borne typhus"[Title/Abstract]) AND ("Acute Febrile Illness"[Title/Abstract] OR "Acute Undifferentiated Febrile Illness"[Title/Abstract] OR "AUFI"[Title/Abstract] OR "pyrexia of unknown origin"[Title/Abstract] OR "PUO"[Title/Abstract] OR "fever"[Title/Abstract]) AND ("Prevalence"[Mesh] OR "Epidemiology"[Mesh] OR "prevalence"[Title/Abstract] OR "incidence"[Title/Abstract] OR "epidemiol*"[Title/Abstract] OR "positivity rate*"[Title/Abstract] OR "seroprevalence"[Title/Abstract] OR "outbreak*"[Title/Abstract] OR "surveillance"[Title/Abstract]) AND ("India"[Mesh] OR "India"[Title/Abstract] OR "Indian"[Title/Abstract] OR "Bharat"[Title/Abstract] OR "Assam"[Title/Abstract] OR "Meghalaya"[Title/Abstract] OR "Shillong"[Title/Abstract])	Broad search strategy: search string includes "AFI" and "PUO" to maximize electronic retrieval sensitivity; Eligibility filter: Studies evaluating broad localized AFI or prolonged PUO without AUFI stratification were systematically excluded during full-text appraisal	420
Embase (Elsevier)	Emtree and proximity syntax: target pathogen, acute undifferentiated fever terms, epidemiological metrics, Indian geographic bounds	('scrub typhus'/exp OR 'orientia tsutsugamushi'/exp OR 'scrub typhus':ti,ab OR 'orientia tsutsugamushi':ti,ab) AND ('acute febrile illness':ti,ab OR 'acute undifferentiated febrile illness'/exp OR 'aufi':ti,ab OR 'pyrexia of unknown origin':ti,ab OR 'fever'/exp) AND ('prevalence'/exp OR 'epidemiology'/exp OR 'incidence':ti,ab OR 'seroprevalence':ti,ab OR 'positivity rate':ti,ab) AND ('india'/exp OR 'india':ti,ab OR 'indian':ti,ab)	Inclusion: peer-reviewed hospital cohorts screening early acute febrile presentations; Exclusion filter: excludes generalized nonundifferentiated febrile series lacking strict baseline triage boundaries	310
The Cochrane Library	Systematic reviews and trials search: Rickettsial disease keywords combined with acute febrile presentation indicators	[mh "Scrub Typhus"] OR [mh "Orientia tsutsugamushi"] OR ("scrub typhus" OR "Orientia tsutsugamushi"):ti,ab,kw AND ("acute febrile illness" OR "AUFI" OR "fever"):ti,ab,kw AND ("prevalence" OR "incidence" OR "epidemiology"):ti,ab,kw AND ("India" OR "Indian"):ti,ab,kw	Inclusion: surveillance registers and screening pools; Exclusion filter: excludes interventional trial protocols lacking baseline observational AUFI denominators	45
Google Scholar	Title-restricted screen: Outbreak tracking, regional hospital series, public health reports	allintitle: "scrub typhus" OR "Orientia tsutsugamushi" "India" OR "Indian" prevalence OR AUFI OR outbreak	Inclusion: institutional screening series and outbreak investigations (first 200 items evaluated); Exclusion filter: narrative reviews, opinion pieces, and case reports (N < 10) excluded	850
Cross-referencing/manual search	Secondary citation mining: Screening reference lists of included articles, reviews, and IDSP bulletins	Manual screening of bibliographies from major rickettsial disease reviews and Indian national surveillance reports	Inclusion: unindexed primary institutional cohorts meeting laboratory confirmation standards; Exclusion filter: asymptomatic community serosurveys and contact clusters excluded	15
Gross total yield	-	-	Cumulative records identified across all electronic search platforms	1,640
Deduplication phase	Reference management and overlap removal	Automated EndNote duplicate removal supplemented by manual title/author verification	Exclusion action: n = 520 duplicate entries removed prior to screening	1,120 unique records remaining
